# Accelerated MR spectroscopic imaging—a review of current and emerging techniques

**DOI:** 10.1002/nbm.4314

**Published:** 2020-05-12

**Authors:** Wolfgang Bogner, Ricardo Otazo, Anke Henning

**Affiliations:** ^1^ High‐Field MR Center, Department of Biomedical Imaging and Image‐Guided Therapy Medical University of Vienna Vienna Austria; ^2^ Department of Medical Physics Memorial Sloan Kettering Cancer Center New York, New York USA; ^3^ Max Planck Institute for Biological Cybernetics Tübingen Germany; ^4^ Advanced Imaging Research Center, UT Southwestern Medical Center Dallas Texas USA

**Keywords:** acceleration, acquisition, compressed sensing, MR spectroscopic imaging, parallel imaging, reconstruction, spatial‐spectral encoding, undersampling

## Abstract

Over more than 30 years in vivo MR spectroscopic imaging (MRSI) has undergone an enormous evolution from theoretical concepts in the early 1980s to the robust imaging technique that it is today. The development of both fast and efficient sampling and reconstruction techniques has played a fundamental role in this process. State‐of‐the‐art MRSI has grown from a slow purely phase‐encoded acquisition technique to a method that today combines the benefits of different acceleration techniques. These include shortening of repetition times, spatial‐spectral encoding, undersampling of *k*‐space and time domain, and use of spatial‐spectral prior knowledge in the reconstruction. In this way in vivo MRSI has considerably advanced in terms of spatial coverage, spatial resolution, acquisition speed, artifact suppression, number of detectable metabolites and quantification precision. Acceleration not only has been the enabling factor in high‐resolution whole‐brain ^1^H‐MRSI, but today is also common in non‐proton MRSI (^31^P, ^2^H and ^13^C) and applied in many different organs. In this process, MRSI techniques had to constantly adapt, but have also benefitted from the significant increase of magnetic field strength boosting the signal‐to‐noise ratio along with high gradient fidelity and high‐density receive arrays. In combination with recent trends in image reconstruction and much improved computation power, these advances led to a number of novel developments with respect to MRSI acceleration. Today MRSI allows for non‐invasive and non‐ionizing mapping of the spatial distribution of various metabolites’ tissue concentrations in animals or humans, is applied for clinical diagnostics and has been established as an important tool for neuro‐scientific and metabolism research. This review highlights the developments of the last five years and puts them into the context of earlier MRSI acceleration techniques. In addition to ^1^H‐MRSI it also includes other relevant nuclei and is not limited to certain body regions or specific applications.

## Graphical abstract

This review summarizes the progress in fast MR spectroscopic imaging (MRSI) of the last five years and puts it into the context of earlier MRSI acceleration techniques. It highlights emerging techniques for time‐efficient sampling of MRSI data including repetition time reduction, spatial‐spectral encoding, *k*‐space undersampling, and use of spatial‐spectral prior knowledge. In addition to ^1^H‐MRSI it also includes other relevant nuclei and is not limited to certain body regions or specific applications.

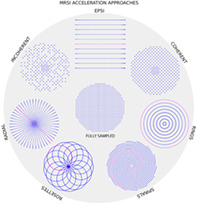

**Abbreviations**
*B*_0_: static magnetic field strength
*B*_1_^+^: transmit RF field
CAIPIRINHA: controlled aliasing in parallel imaging results in higher acceleration
CRT: concentric ring trajectory
CS: compressed sensing
CSDE: chemical shift displacement error
EPI: echo planar imaging
EPSI: echo‐planar spectroscopic imaging
FID: free induction decay
FIDLOVS: FID acquisition localized by outer volume suppression
GRAPPA: generalized autocalibrating partially parallel acquisition
GSLIM: generalized series approach to MR spectroscopic imaging
IDEAL: iterative decomposition of water and fat with echo asymmetry and least‐squares estimation
LASER: localization by adiabatic selective refocusing
MRSI: MR spectroscopic imaging
OVS: outer volume suppression
PEPSI: proton EPSI
PI: parallel imaging
PRESS: point resolved spectroscopy
SAR: specific absorption rate
SBW: spectral bandwidth
SE: spin echo
SENSE: sensitivity encoding
SLAM: spectroscopy with linear algebraic modeling
SLIM: spectral localization by imaging
SLOOP: spectral localization with optimal pointspread function;
SMS: simultaneous multi‐slice
SNR: signal‐to‐noise ratio
SNR/*t*: SNR per unit time
SPICE: spectroscopic imaging by exploiting spatiospectral correlation
SRF: spatial response function
SSE: spatial‐spectral encoding
SSFP: steady‐state free precession
*T*_E_: echo time
*T*_R_: repetition time
UHF: ultra‐high field

## 1 INTRODUCTION

Shortly after the introduction of in vivo MRI and non‐localized in vivo MRS, in vivo MR spectroscopic imaging (MRSI) was demonstrated in the early 1980s. Two distinct acquisition methods were suggested: (i) “NMR chemical shift imaging in three dimensions” in 1982 by Brown et al.^1^ and “Spatially resolved high‐resolution spectroscopy by 4‐dimensional NMR” by Maudsley et al.^2^ in 1983 as well as (ii) “Spatial mapping of the chemical shift in NMR” in 1984 by Mansfield.^3^ While (i) corresponds to classical phase‐encoded and thus non‐accelerated MRSI, (ii) represents the very first description of the most popular spatial‐spectral MRSI acceleration method, echo‐planar spectroscopic imaging (EPSI).^4^ In these early days hardware limitations such as gradient strength and fidelity as well as static magnetic field strength (*B*_0_) and homogeneity hindered the translation of these theoretical concepts into the excellent results that we can obtain today. However, during the last 30 years in vivo MRSI has undergone an enormous evolution in terms of spatial coverage, spatial resolution, acquisition speed, artifact suppression, number of detectable metabolites, and quantification accuracy and precision. Today it allows for non‐invasive and non‐ionizing whole‐organ (e.g., brain) mapping of various metabolites’ tissue concentrations in animals and humans (e.g., up to 12 brain metabolites^5^ and nine macromolecular compounds^6^), up to 300 times faster than conventional phase‐encoded ^1^H‐MRSI.^7^ While mainly conventional point resolved spectroscopy (PRESS)‐localized Cartesian‐sampled MRSI is currently applied for clinical diagnostics,^8^ basic and advanced MRSI protocols have been established as important tools for neuro‐scientific and metabolism research.^8^

Due to the need to encode the chemical shift along with spatial information, classical MRSI utilizes phase encoding along all directions, which is intrinsically slow. Another complication is the very low signal intensity of metabolites. In comparison to ^1^H‐MRI of tissue water, ^1^H‐MRSI of tissue metabolites is about 10 000 times less sensitive due to the concentration difference between water and metabolites. In addition, abundant water and fat signals lead to much stricter needs to control related artifacts. MRSI of other nuclei such as ^31^P, ^2^H or ^13^C is yet another order of magnitude less sensitive and lacks the possibility to use anatomical images as reference and to generate prior knowledge, which further restricts the acceleration options. Hyperpolarized ^13^C‐MRSI has sufficiently high signal‐to‐noise ratio (SNR) and only a few metabolite peaks, but suffers from acquisition time restrictions. Thus, hyperpolarized ^13^C‐MRSI has benefitted particularly from the development of fast MRSI encoding techniques in the past, achieving nowadays time resolutions needed for assessing real‐time metabolic fluxes in clinical settings.^9^

While the basic principles of ^1^H‐MRI acceleration are applicable to ^1^H‐ and non‐proton MRSI as well, they usually need substantial adaptation to encode chemical shift, work robustly in a low‐SNR regime and in the presence of strong nuisance signals (e.g., water, fat) or meet the scan time restrictions in hyperpolarized ^13^C‐MRSI.

There are four main principles for accelerating MRSI: (i) short repetition times (*T*_R_), (ii) acquisition of multiple *k*‐space points per *T*_R_, (iii) *k*‐space undersampling and (iv) data reconstruction using spectral or spatial prior knowledge. Reports on the short‐*T*_R_ principle range from moderately short *T*_R_ values to true steady‐state free precession (SSFP). The acquisition of multiple *k*‐space points per *T*_R_ can be accomplished by either multi‐spin‐echo sampling or more commonly spatial‐spectral encoding (SSE) including EPSI or non‐Cartesian *k*‐space trajectories. In a broad sense “*k*‐space undersampling” methods include widely available methods such as elliptical *k*‐space shuttering^10^ or MRS pre‐localization (i.e., allowing field‐of‐view reduction with fewer required *k*‐space points). “Real” *k*‐space undersampling approaches include parallel imaging (PI), multi‐band acquisition, and compressed sensing (CS). Finally, there are reconstruction techniques that use prior knowledge from high‐resolution anatomical images and spectral priors, and combinations with low‐rank reconstruction. All these acceleration principles are complementary and can be combined. A detailed introduction of *k*‐space formalism and filtering is outside the scope of this review. We refer the interested reader to two excellent books that provide a general introduction, before continuing with the more specific sections detailed below.^10^, ^11^

Related MRSI encoding methods have been reviewed by a number of previous review papers and book chapters^12^, ^13^, ^14^, ^15^, ^16^ that either cover mostly literature from before 2014 or have a limited coverage with respect to acceleration methods or applications. Recent progress in MRSI encoding has significantly benefitted from ultra‐high field (UHF) systems, high gradient fidelity and high‐density receive coil arrays. In addition, a number of dedicated acceleration methods for hyperpolarized ^13^C‐MRSI have been demonstrated. In combination with emerging trends in image reconstruction and much improved computation power, these advances led to a number of novel developments with respect to MRSI acceleration. Hence, this review highlights the developments of the last 5 years and puts them into the context of earlier techniques. In addition to ^1^H‐MRSI it also includes other relevant nuclei and is not limited to certain body regions or specific applications. Thus, this review paper represents the most comprehensive and most up to date one of its kind.

## 2 SHORT *T*_R_/*T*_E_

The most straightforward way to speed up MRSI is by either shortening the *T*_R_,^17^, ^18^, ^19^ or acquiring multiple spin echoes (SEs) per *T*_R_.^14^, ^15^ This approach has its roots in the early 2000s, when highly SNR‐efficient SSFP as well as turbo‐SE sequences were proposed for MRSI.^17^, ^18^, ^19^, ^20^, ^21^ These sequences benefit either from fast point‐by‐point sampling of the *k*‐space with short *T*_R_,^17^, ^18^, ^19^ or from sampling of multiple *k*‐space points per *T*_R_ in the case of multiple SEs^20^, ^21^ (ie, their relative contribution is a function of the *T*_1_/*T*_2_ ratio and flip angles). Both methods restrict the acquisition time for the spectral readout to the inter‐pulse delay. Due to the resulting reduced spectral resolution,^21^, ^22^ SSFP‐MRSI was initially confined to well separated resonances (e.g., fat/water mapping, only the singlets of NAA, Cr and Cho in ^1^H‐MRSI, or ^31^P/^13^C‐MRSI)^19^, ^23^, ^24^ and animal studies (i.e., featuring faster encoding gradients and higher spectral resolution due to available high‐*B*_0_ MR scanners).^17^, ^18^ This changed when improved hardware became available also for human whole‐body MR systems and enabled the acquisition of true SSFP‐based ^1^H‐MRSI (*T*_R_ < 200 ms) of the human brain with sufficiently high spatial and spectral resolution.^25^, ^26^, ^27^ Although SSFP‐based MRSI yields the highest possible SNR efficiency, major downsides include the low spectral resolution, limited water suppression, potential banding artifacts and strongly *T*_1_‐/*T*_2_‐weighted metabolite signals, which make metabolic maps highly qualitative without relaxation corrections, and interpretation of the exact neurochemical underpinnings in pathologies challenging. Recent implementations overcome problems with banding artifacts by using balanced SSFP with either a very narrow pass‐band frequency selective excitation and frequency sweeping^28^ or successive measurements with phase increments to achieve the spectral separation in a similar manner to the Dixon approach^29^

**FIGURE 1:**
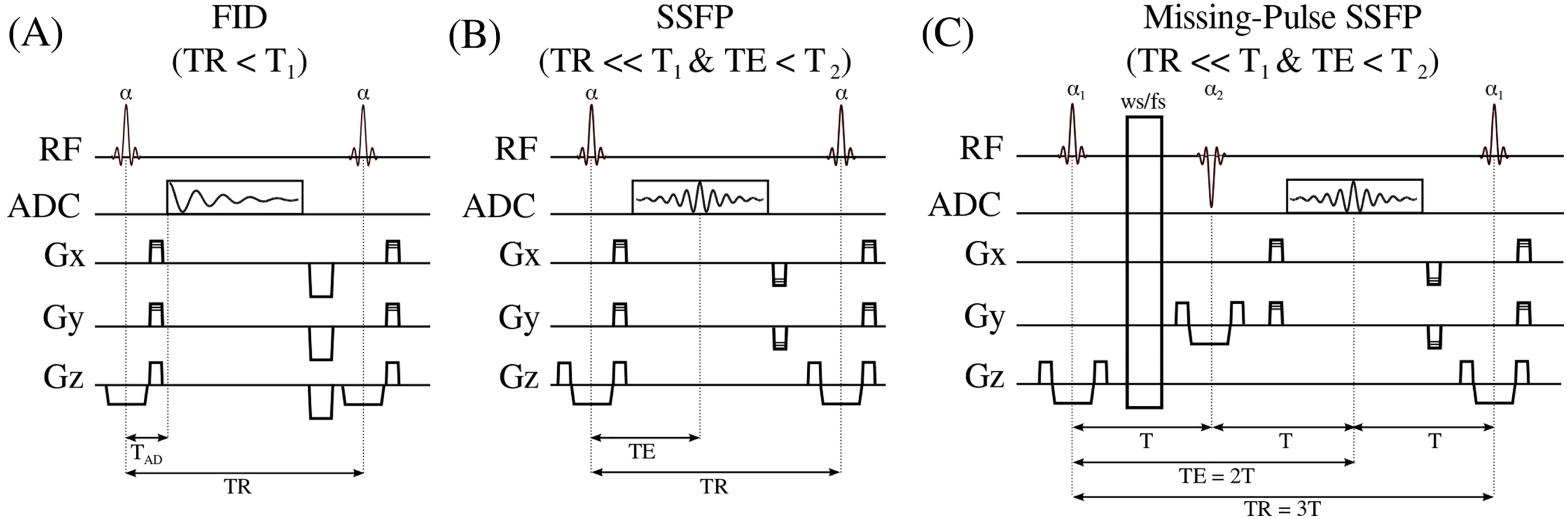
Comparison of different MRSI sequence types that make use of short *T*_R_ values for acceleration. A, FID‐MRSI sequences acquire an FID signal after an Ernst excitation pulse followed by a gradient spoiler to eliminate any remaining transverse magnetization. B, C, In contrast, SSFP‐MRSI sequences revert the phase‐encoding after signal sampling, so that the remaining transversal magnetization can be reused and the steady‐state condition can be established. Using this magnetization the next RF pulse creates an echo. This transversal magnetization continues to be refocused until the signal decays to zero. Therefore, each sampling interval collects the sum signal of multiple echoes, the relative contributions of which are determined by their *T*_2_. Because of the short *T*_R_, the signal is additionally *T*_1_ weighted. The mix of *T*_1_/*T*_2_‐weighting is specific for each resonance (not metabolite) and depends also on the local excitation flip angle. C, As a more robust alternative missing‐pulse SSFP has been proposed to allow detection of complete SEs (eliminating the need for phasing, mitigating truncation artifacts and improving spectral resolution) and enabling the incorporation of improved spatial localization as well as water/fat suppression pulses. Courtesy of Philipp A. Moser

**FIGURE 2:**
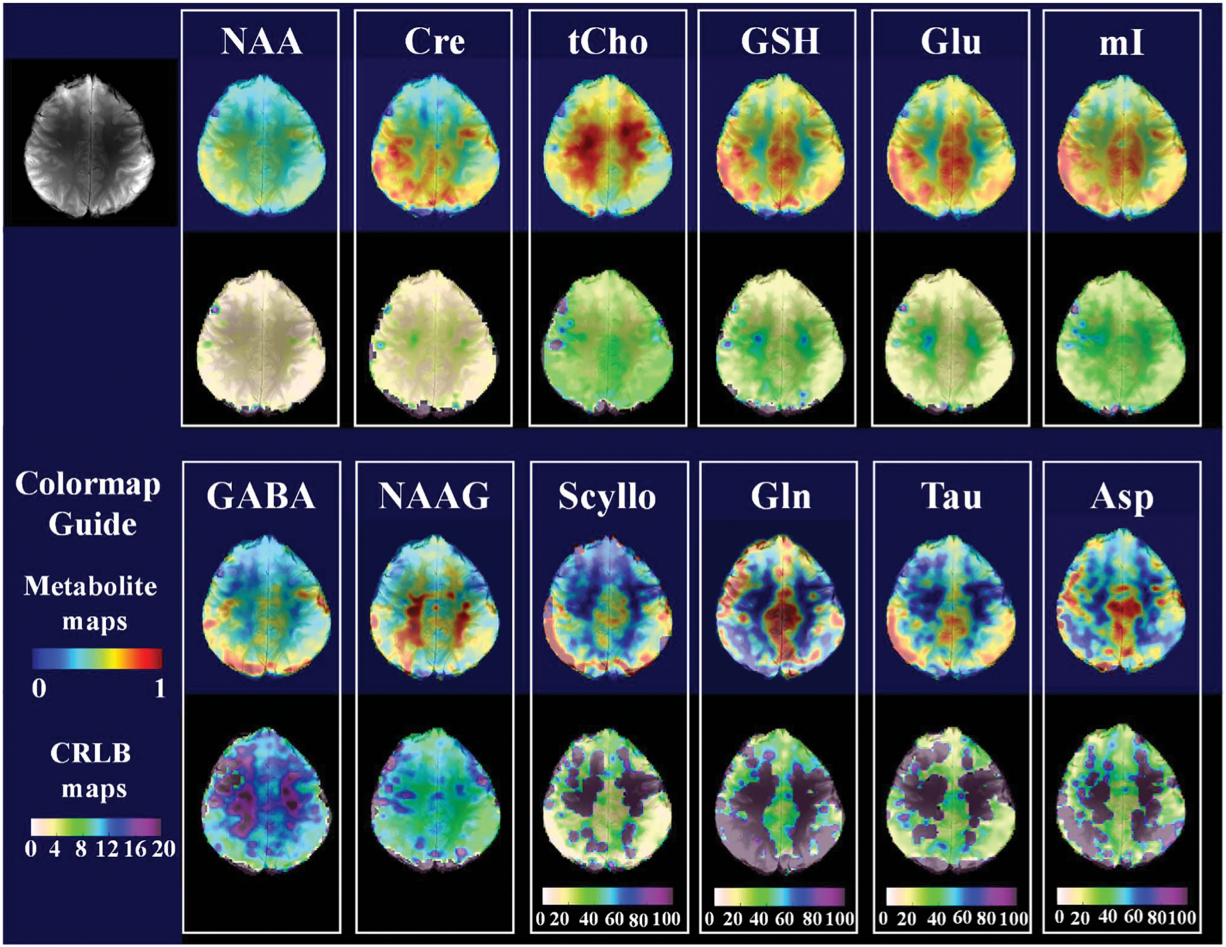
Ultra‐high resolution (128 × 128) metabolite maps for 12 different metabolites along with the anatomical reference scan (top left) obtained via FID‐MRSI at 9.4 T using a short *T*_R_ of 220 ms and acquisition delay of 1.5 ms. The CRLB maps are shown below the metabolite map in each box. Note the different scaling of the CRLB maps in the last four metabolites (Scyllo, Gln, Tau and Asp). Reproduced from the work of Nassirpour et al^5^

Albeit not as SNR efficient as SSFP‐MRSI, the lack of undesired *T*_2_‐weighting or *J*‐coupling effects has made pulse‐acquire or free induction decay (FID)‐MRSI sequences with Ernst angle excitation and gradient spoiling after each *T*_R_ (Figure 1) an increasingly popular alternative, not only for ^31^P‐MRSI^30^, ^31^, ^32^, ^33^, ^34^ and ^13^C‐MRSI,^24^, ^35^, ^36^, ^37^, ^38^ but also recently for ^1^H‐MRSI.^5^, ^39^, ^40^, ^41^, ^42^ The FIDLOVS (FID acquisition localized by outer volume suppression) sequence^43^ had still a fairly complicated design with a large number of outer volume suppression (OVS) pulses causing high specific absorption rate (SAR) demands at 7 T and consequently long *T*_R_ values, but it motivated simpler ^1^H‐FID‐MRSI approaches that allowed for much shorter *T*_R_ values of a few hundred milliseconds by confining the sequence to water suppression and excitation pulses.^39^, ^44^ The *T*_R_‐shortening of this type of sequence is only limited by a possible compromise in water suppression quality, *T*_1_‐weighting and spectral resolution (a side‐effect of reducing the FID sampling). Some implementations have even completely abandoned all suppression pulses and replaced these by retrospective nuisance removal.^45^ In particular at UHF ^1^H‐FID‐MRSI overcomes critical limitations in SAR, chemical shift displacement error (CSDE), *B*_1_^+^‐inhomogeneity, *T*_2_‐weighting, *J*‐evolution and coverage of cortical regions in comparison with traditional MRSI. On the other hand, ^1^H‐FID‐MRSI lacks high‐quality volume selection to reduce nuisance signals (e.g., extracranial lipids) and macromolecule signals are enhanced. While this makes accurate metabolite quantification challenging, it has enabled the high‐resolution mapping of macromolecules.^6^, ^46^, ^47^, ^48^ Since then, FID‐MRSI acquisitions with short *T*_R_ values (~60 to 600 ms) have become an integral part of many UHF ^1^H‐MRSI sequences at 7 T (References 41, 48, 49, 50, 51, 52, 53) and 9.4 T (References 5, 40, 54, 55, 56, 57) (Figure 2) and an increasing number of 3 T MRSI implementations.^51^, ^54^, ^58^ FID‐MRSI is also the method of choice for ^31^P‐MRSI^30^, ^31^, ^32^, ^33^, ^34^ due to the fast *T*_2_ relaxation of most ^31^P‐MRS signals, and for ^2^H‐MRSI^59^ due to the absence of water and fat suppression modules. FID‐MRSI applications are so far limited mostly to the human brain but also for the investigation of skeletal muscles and ^31^P‐MRSI,^30^, ^42^ where volume pre‐selection is secondary. First clinical examples have been demonstrated in patients with brain tumors,^7^, ^31^, ^49^ multiple sclerosis^7^, ^50^, ^60^ and mild cognitive impairment.^61^, ^62^

In principle, also SE‐MRSI sequences can be used with shorter *T*_R_, but this is uncommon, since SSFP‐MRSI is more efficient and especially the high bandwidth and SAR demands of refocusing pulses tailored for UHF (e.g., adiabatic RF pulses in particular) are a major constraint.

While the acquisition time saving associated with *T*_R_ reduction does not match that of common SSE approaches, the achievable acceleration factors are nevertheless good (~2‐ to 20‐fold) compared with typical undersampling techniques and come with only minor side effects (e.g., *T*_1_‐weighting, possible FID truncation), while benefitting among other advantages from a slight boost in SNR per unit time (SNR/*t*).^39^ Combinations with other acceleration methods, which all have the tendency to reduce SNR efficiency, are straightforward. Successful combinations have been shown with PI,^41^, ^51^, ^52^, ^55^, ^56^ CS, ^57^ SSE^63^, ^64^ or several approaches at once to enable high‐resolution rapid whole‐brain MRSI^7^, ^64^ or dynamic 2D‐MRSI with high temporal resolution.^65^

## 3 SPATIAL‐SPECTRAL ENCODING

Even higher accelerations can be achieved using SSE. The basic principle of SSE was introduced by Mansfield^3^ in 1984, but early implementations were limited by gradient hardware imperfections.^66^, ^67^ It took decades until SSE developed into the robust metabolic imaging tool that it is today.^68^, ^69^, ^70^, ^71^

In contrast to phase‐encoded MRSI, where spatial and spectral encoding are strictly separated, SSE utilizes high‐slew‐rate gradient waveforms to sample spatial information (by sampling along *k*‐space trajectories) simultaneously with spectral information (by repeating the same trajectory a few hundred times per *T*_R_) (Figure S1). This is possible since the encoding of the spectral dimension (sampling period in ms) is a slow process compared with the encoding of spatial dimensions (sampling period in μs). SSE enables highly efficient and about 25 to 170 times faster MRSI scans than pure phase‐encoding.^72^ The SNR/*t* is similar to that of conventional phase‐encoding, provided that the most efficient sampling (e.g., ramp sampling for EPSI) is used.^73^, ^74^

**FIGURE 3:**
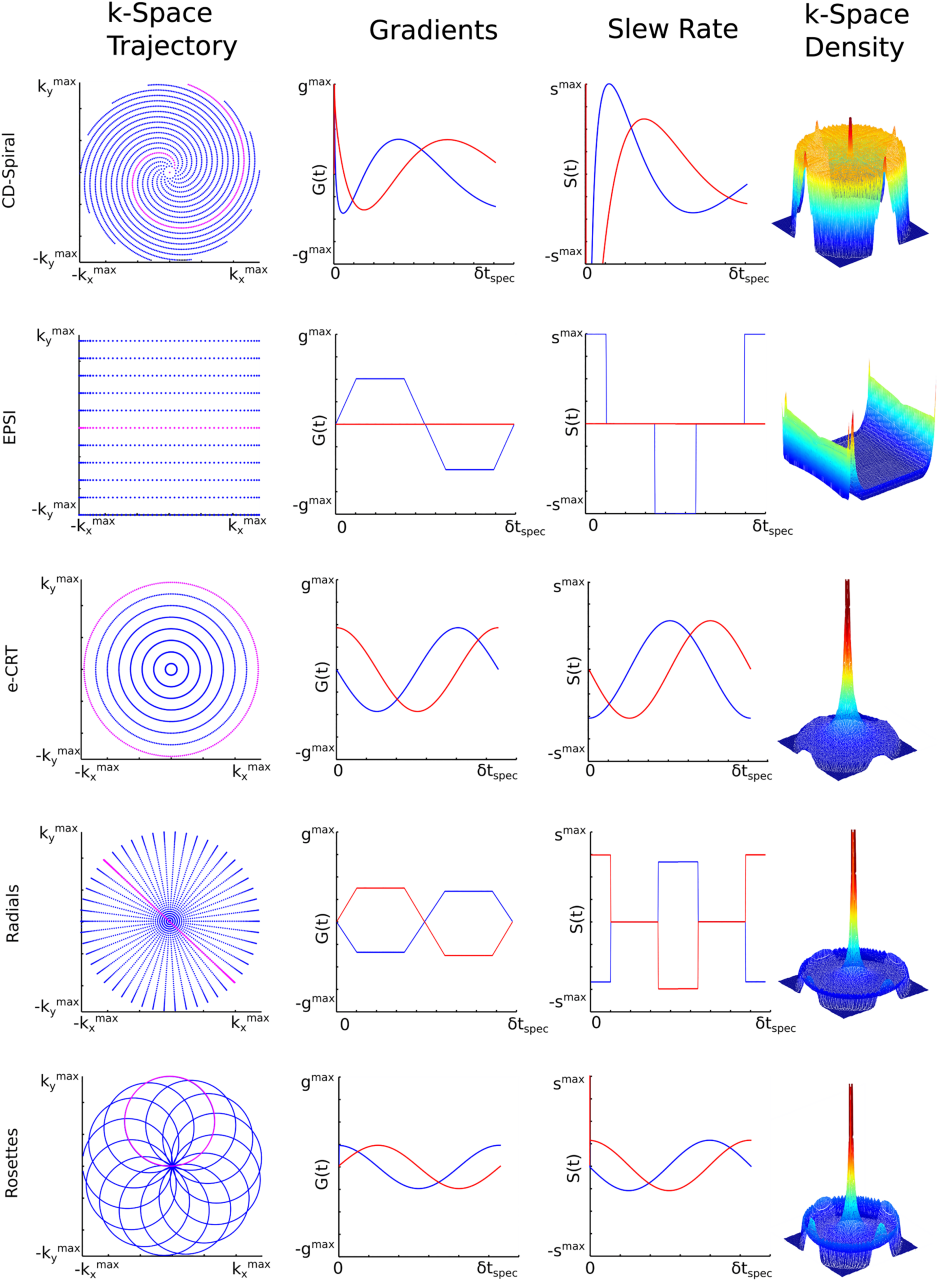
Qualitative side‐by‐side comparison of the most common SSE strategies (i.e., constant‐density spirals, symmetric EPSI, equidistant CRT, radial EPSI and rosettes) for encoding of a 2D *k*‐space (left column). In all cases a single *k*‐space trajectory, which is repeated several hundred times for a single *T*_R_, is highlighted in pink while all remaining trajectories to fill the *k*‐space are displayed in blue. The time evolution of gradient strength and slew rate are displayed for *x*‐ and *y*‐gradients (blue and red, respectively) (middle columns). The achieved *k*‐space density as derived by simulations is illustrated by color‐coded surface plots. Courtesy of Lukas Hingerl

**FIGURE 4:**
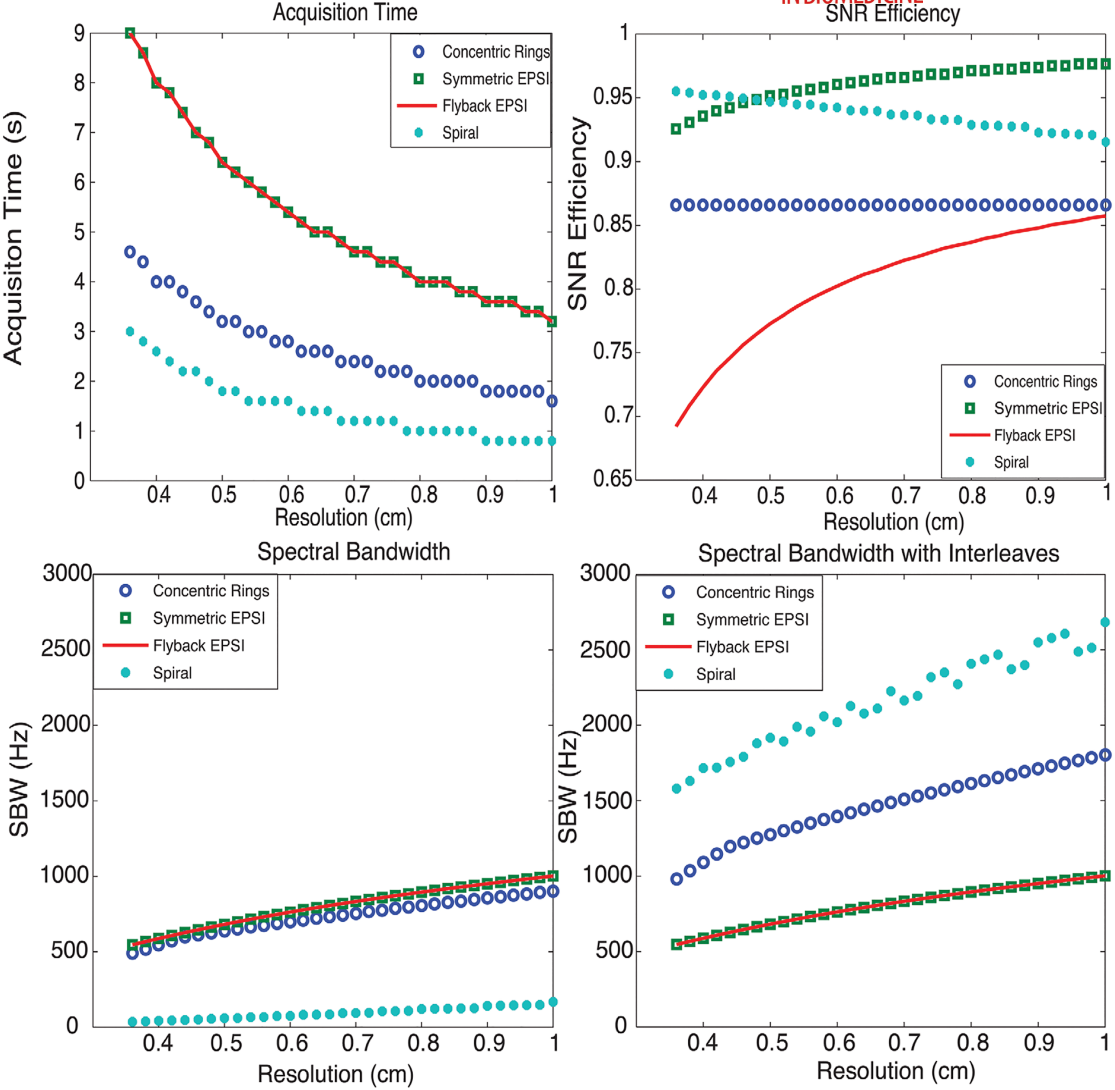
Comparison of concentric rings, EPSI and spiral spectroscopic imaging: top left, acquisition time; top right, SNR efficiency; bottom left, bottom right, SBW and SBW with spectral interleaves. CRT requires half of the total acquisition time compared with EPSI trajectories, offers about 87% SNR efficiency and provides much wider spectral bandwidth than flyback‐EPSI and symmetric EPSI. Although nominally spirals are the most efficient trajectories, offering the best acquisition time and SBW benefit while sacrificing the least SNR, they are limited by susceptibility to gradient infidelities. All designs assumed a gradient amplitude limit of 40 mT/m and maximum slew rate of 150 mT/m/ms. Top right, SNR efficiency of different trajectories for various resolutions. The SNR loss for flyback EPSI is mostly due to its low duty cycle. The finer the resolution is, the lower the duty cycle will be, and SNR efficiency decreases as the flyback portion requires more time. Although the duty cycle for symmetric EPSI with ramp sampling is 100%, the non‐uniform ramp sampling reduces SNR efficiency. For the constant slew rate spiral trajectories, SNR efficiency decreases as the resolution becomes coarser with a fixed FOV, since there is proportionally less outer *k*‐space sampling where spirals are more uniform. Non‐uniformity causes most of the SNR loss of spirals, while duty cycle results in a smaller fraction of the loss. Benefitting from the design of constant slew rate, the spiral trajectories provide even better SNR efficiency than flyback EPSI and CRT. CRT offers a constant SNR efficiency, which is better than flyback EPSI with the chosen prescriptions. The loss of SNR efficiency for CRT is caused by the non‐uniformity. Note that the SNR efficiency depends on the targeted *k*‐space density. Here a constant density *k*‐space was targeted. Given the same traversing velocity, the achieved SBWs for EPSI, CRT and spirals are decreasing (bottom left) without interleaves. To exploit the maximum SBW, both symmetric EPSI and flyback EPSI result in the same waveform, thus achieving the same SBW. They are only slightly better than CRT, since flyback EPSI requires flyback time and symmetric EPSI does not critically exploit the whole SBW. However, CRT and spiral trajectories are more scan‐time efficient compared with EPSI. If we take advantage of scan‐time efficiency by applying temporal interleaves, we can increase SBW. Bottom right, SBW of all trajectories was computed by accounting for the temporal interleaves constrained for the same total acquisition time. For this tradeoff, spirals offer the best SBW, while CRT's SBW is doubled compared with EPSI. The non‐monotonicity of the spiral trajectory SBW with respect to resolution in this analysis is due to using an integer number of interleaves. Reproduced from the work of Jiang et al.^86^

SSE can employ a variety of different Cartesian or non‐Cartesian trajectories that have very different gradient requirements and *k*‐space densities (Figure 3), with EPSI^75^ and spiral‐based MRSI^76^ being the most prominent examples. However, gradient hardware limitations and the need for higher spectral bandwidth (SBW)/spatial resolution at higher *B*_0_,^73^, ^77^, ^78^ as well as the desire to reduce voxel bleeding by an improved spatial response function (SRF) (i.e., to mitigate extracranial lipid artifacts), have made alternative SSE strategies increasingly attractive^79^, ^80^, ^81^, ^82^ Overall, the use of SSE becomes challenging at UHF (i.e., the SNR efficiency suffers) since the maximum time permitted for one trajectory repetition (i.e., spectral dwelltime) is inverse proportional to the SBW, and trajectory repetitions become challenging for the higher spatial resolutions at UHF (Figure 4). Although temporal interleaving can somewhat alleviate this otherwise hard limit at the cost of measurement speed, the number of temporal interleaves is limited to three or less for ^1^H‐MRSI, while it is freely adjustable for multi‐nuclear MRSI.^7^, ^30^, ^63^, ^83^ The reason is that for more than three temporal interleaves high unsuppressed water signal lead to spectral aliasing artifacts in the spectral range of interest (e.g., between water and lipid signals), the intensity of which scales with temporal instabilities between temporal interleaves and cannot be easily removed.^15^ A completely different approach is reduction of acquisition time using partition of the signal decay in spectroscopic imaging (RAPID‐SI),^84^which is similar to an approach shown earlier by Cao et al for hyperpolarized ^13^C‐MRSI.^85^ Both speed up MRSI acquisition along a phase‐encoding direction approximately 2‐ to 16‐fold by separating the acquired FID into equidistant fractions via blipped phase‐encoding gradients (this resamples jumping from one *k*‐space point to the next during FID readout rather than moving along a continuous trajectory).

**FIGURE 5:**
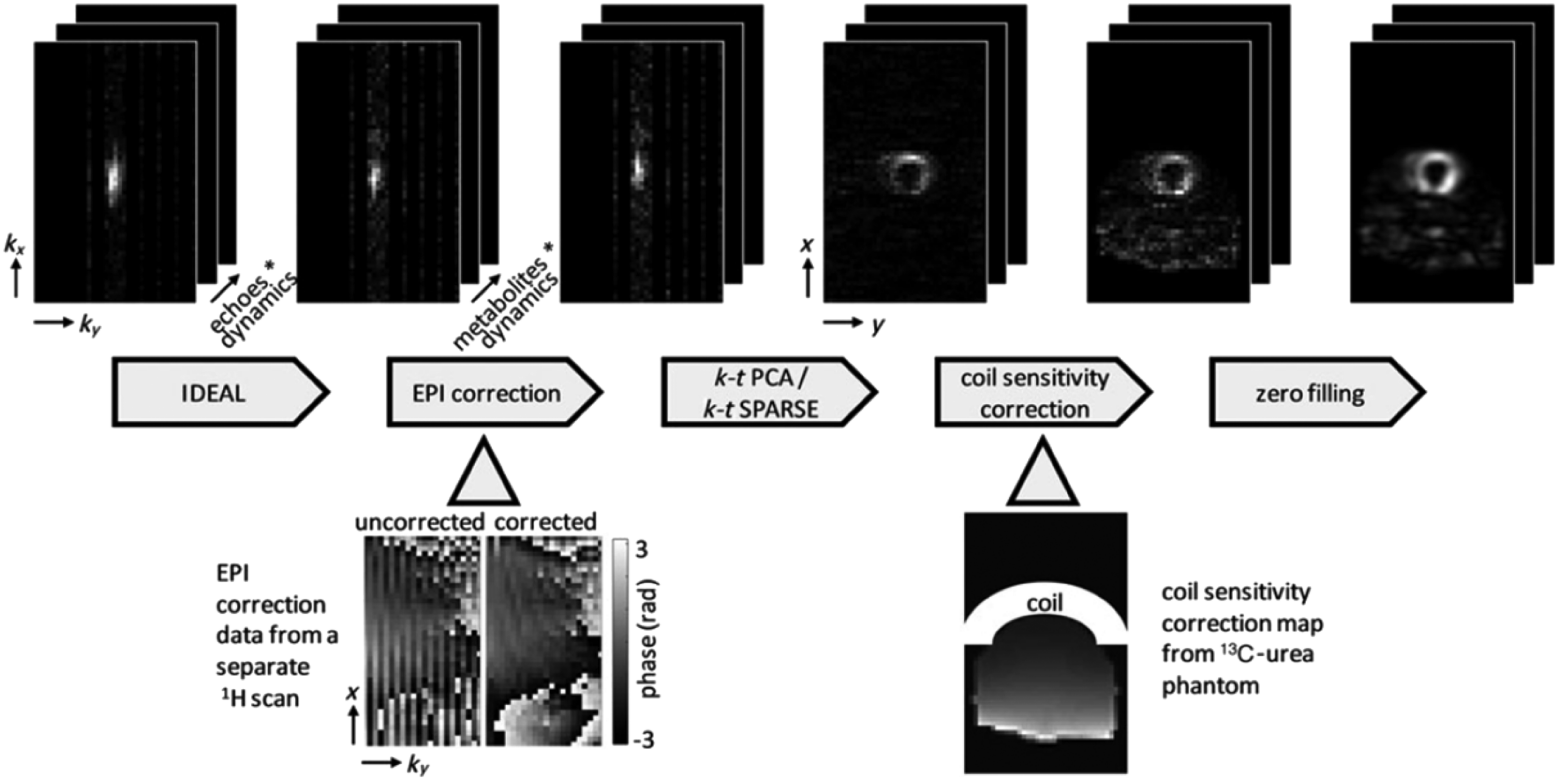
Diagram of the ^13^C data reconstruction steps with intermediate results. The IDEAL reconstruction transforms the seven echoes per dynamic into the five metabolites (pyruvate, lactate, bicarbonate, pyruvate hydrate and alanine) per dynamic. EPI ghost correction is performed using additionally acquired ^1^H data. *k*‐*t* principal component analysis (*k*‐*t* PCA) or *k*‐*t* CS (*k*‐*t* SPARSE) is then applied and the data are transformed to image space. A surface coil sensitivity correction map acquired from a ^13^C‐urea phantom is applied to compensate for the reduced sensitivity in the inferior segment of the heart. Finally, the data are zero‐filled. The example images show data from a *k*‐*t* PCA scan and the lactate signal of a single dynamic. Reproduced from the work of Wespi et al^93^

Since EPSI is in principle equivalent to multi‐echo MRI with extremely short echo spacing (i.e., spectral dwelltime in MRSI), Dixon‐based spectral separation can be considered an extreme case of SSE with very few (i.e., originally two), sometimes non‐equidistantly distributed, echoes. To quantify a few well‐separated spectral resonances (e.g., water and fat^87^, ^88^ or four resonances in ^13^C‐MRSI^89^), only a few echoes (i.e., EPSI lines or any other SSE trajectories) must be acquired. The basic idea is to exploit the difference in precession frequency between two or multiple resonances. For instance, to separate fat and water, two images with slightly different *T*_E_ values are acquired. For the first image the *T*_E_ is adjusted to show fat and water signals in phase, while the *T*_E_ for the second image is adjusted by a few milliseconds to have fat and water signals out of phase. Adding the two images leads to a water image and subtracting the two images to a fat image. The Dixon method and the related iterative decomposition of water and fat with echo asymmetry and least‐squares estimation (IDEAL) can be extended to the separation of multiple resonances by using multiple spin or gradient echoes. IDEAL has been combined with spiral and echo planar imaging (EPI) readouts as well as *k*‐*t* undersampling approaches for hyperpolarized ^13^C‐MRSI^89^, ^90^, ^91^, ^92^, ^93^ (the processing pipeline is shown in Figure 5) and ^129^Xe‐MRSI.^94^

Apart from the maximum possible acceleration factor and SNR efficiency, a main criterion for choosing the right SSE technique is the limitations imposed by the available gradient system. The targeted spatial resolutions are much lower in MRSI than in MRI. Hence, maximum gradient amplitudes do not impose any limitations, but a high gradient slew rate is critical to allow gradient trajectories to remain short and be repeated rapidly. This is necessary to cover the target spectral range (increasing with *B*_0_) and traverse a sufficiently large *k*‐space (otherwise limiting spatial resolution).^63^ The extent to which different SSE trajectories are susceptible to or induce gradient imperfections varies substantially. Generally, *k*‐space trajectory errors are more problematic for spirals, rosettes and radial EPSI than for other SSE alternatives^67^, ^86^, ^95^, ^96^ (see below and Table 1), but these deviations between actual and real trajectories can be measured and considered during MRSI reconstruction.^67^

**TABLE 1:** List of major MRSI acceleration methods (without combinations). Methods listed in different categories can be combined. We differentiate three ranges of acceleration (~1.5 to 8, low; ~8 to 20, moderate; 20 and above, high) and encoding methods that additionally increase (SNR/*t* gain) or decrease (SNR/*t* loss) SNR per time efficiency as compared against a current clinical standard ^1^H‐MRSI protocol (Cartesian sampling, *T*_R_ = 1500 ms, *T*_E_ = 30 ms), respectively

Category	Method	Pros	Cons	Application
Short‐*T*_R_/*T*_E_	SSFP	‐ highest SNR/*t* gain ‐ moderate acceleration	‐ *T*_1_/*T*_2_‐weighting ‐ low spectral resolution ‐ poor water and lipid suppression in ^1^H‐MRSI ‐ banding artifacts/*B*_0_ sensitive	‐ metabolites with long *T*_2_ and short *T*_1_ ‐ hyperpol. ^13^C MRSI/MRI ‐ ^1^H‐MRSI possible but restricted to major singlets ‐ preferably <7 T
	Turbo‐spin‐echo	‐ low acceleration	‐ low spectral resolution ‐ *T*_2_‐weighting (in *k*‐space) ‐ Δ*B*_1_^+^ sensitive	‐ metabolites with long *T*_2_ ‐ singlets in ^1^H MRSI ‐ preferably <3 T
	FID‐MRSI	‐ SNR/*t* gain ‐ moderate acceleration ‐ high SNR ‐ *J*‐coupled metabolites in phase ‐ Δ*B*_1_^+^ insensitive ‐ low SAR ‐ low CSDE	‐ *T*_1_‐weighting ‐ trade‐off between speed (*T*_R_) and spectral resolution ‐ moderate lipid suppression in ^1^H‐MRSI	‐ short *T*_2_/*J*‐coupled metabolites ‐ ultra‐high field ‐ ^13^C/^31^P/^1^H‐MRSI ‐ preferably >1.5 T
Cartesian SSE	EPSI	‐ high acceleration ‐ inherently constant *k*‐space weighting	‐ some SNR/*t* loss ‐ limited SBW/spatial resolution ‐ gradient demanding	‐ ^13^C/^31^P/^1^H‐MRSI ‐ preferably <7 T
Non‐Cartesian SSE	Spirals	‐ highest acceleration ‐ any *k*‐space weighting possible	‐ some SNR/*t* loss ‐ limited SBW/spatial resolution ‐ gradient demanding	‐ ^13^C/^31^P/^1^H‐MRSI ‐ preferably <7 T
	CRTs	‐ high acceleration ‐ inherent *k*‐space weighting (optimization possible)	‐ some SNR/*t* loss ‐ limited SBW/spatial resolution ‐ gradient demanding	‐ ^13^C/^31^P/^1^H‐MRSI ‐ preferably ≥3 T
	Rosettes	‐ can be tailored for either high speed or low gradient stress ‐ inherently weighted *k*‐space (optimization possible)	‐ some SNR/*t* loss ‐ moderate SBW/spatial resolution limitation	‐ ^13^C/^31^P/^1^H‐MRSI ‐ preferably ≥7 T
	Radial EPSI	‐ high acceleration ‐ inherent *k*‐space weighting (fixed)	‐ some SNR/*t* loss ‐ limited SBW/spatial resolution ‐ gradient demanding	‐ ^13^C/^31^P/^1^H‐MRSI ‐ preferably <7 T
Coherent *k*‐space undersampling	SENSE	‐ no gradient demands ‐ low acceleration	‐ some SNR/*t* loss ‐ needs multi‐channel receive coils ‐ needs explicit sensitivity maps ‐ spatial aliasing ‐ motion sensitive	‐ preferably ^1^H‐MRSI ‐ ^13^C/^31^P‐MRSI possible, but difficult to obtain reliable sensitivity maps ‐ preferably ≥3 T/better at UHF
	GRAPPA	‐ no gradient demands ‐ interleaving to reduce motion sensitivity ‐ low acceleration	‐ some SNR/*t* loss ‐ needs multi‐channel receive coils ‐ spatial aliasing	‐ preferably ^1^H‐MRSI ‐ ^13^C/^31^P‐MRSI possible ‐ preferably ≥3 T/better at UHF
	CAIPIRINHA	‐ no gradient demands ‐ better control of aliasing ‐ interleaving to reduce motion sensitivity ‐ low acceleration	‐ some SNR/*t* loss ‐ needs multi‐channel receive coils ‐ spatial aliasing	‐ preferably ^1^H‐MRSI ‐ ^13^C/^31^P‐MRSI possible ‐ preferably ≥3 T/better at UHF
Multi‐slice excitation	Multi‐band/SMS	‐ accelerate also in slice direction ‐ low acceleration	‐ some SNR/*t* loss ‐ needs multi‐channel receive coils ‐ increased SAR/*B*_1_^+^ ‐ spatial aliasing	‐ preferably ^1^H‐MRSI, but ^13^C/^31^P‐MRSI possible ‐ better at UHF
Incoherent *k*‐space undersampling	CS	‐ SNR/*t* gain through regularization ‐ moderate acceleration	‐ sparse data (representation) required ‐ minimum SNR required to work robustly	‐ in spectral domain only for long‐*T*_E_^1^H‐MRSI or ^13^C/^31^P‐MRSI
Prior knowledge based	SLIM/SLOOP/SLAM	‐ SNR/*t* gain through regularization and spatial averaging ‐ high acceleration	‐ sensitive to bias fields such as *B*_0_ inhomogeneity	‐ ^31^P‐MRS(I) ‐ potentially hyperpol. ^13^C‐MRS(I) ‐ spectra from multiple arbitrarily shaped compartments instead of metabolite maps (except for GSLIM)
	SPICE	‐ SNR/*t* gain through regularization ‐ high acceleration	‐ requires assumptions about spatial and spectral priors; nuisance removal challenging ‐ may lead to spatial averaging ‐ may lead to spectral information loss	‐ preferably sparse well resolved spectra (^13^C, ^31^P), but ^1^H‐MRSI possible
	Super‐resolution reconstruction	‐ resolution increase via pure post‐processing	‐ requires assumptions about spatial priors ‐ no true spatial resolution gain—only smoother appearance of metabolite maps	all
	Spectral‐spatial excitation & IDEAL	‐ replaces time‐consuming spectral encoding by conventional MRI readout	‐ requires good spectral separation and Δ*B*_0_ homogeneity	‐ ^13^C/^31^P‐MRSI; good spectral separation required

Abbreviation: Δ*B*_1_^+^—transmit field inhomogeneity.

### 3.1 Cartesian SSE

Cartesian SSE techniques acquire one spatial dimension and the spectral dimension simultaneously in a single readout via a series of periodically inverted readout gradients. Each semi‐period encodes one line in *k*‐space and the progression of gradient pulses encodes the spectral dimension. Symmetric‐EPSI and flyback‐EPSI are most commonly used (Figure S1).

#### 3.1.1 EPSI

**FIGURE 6:**
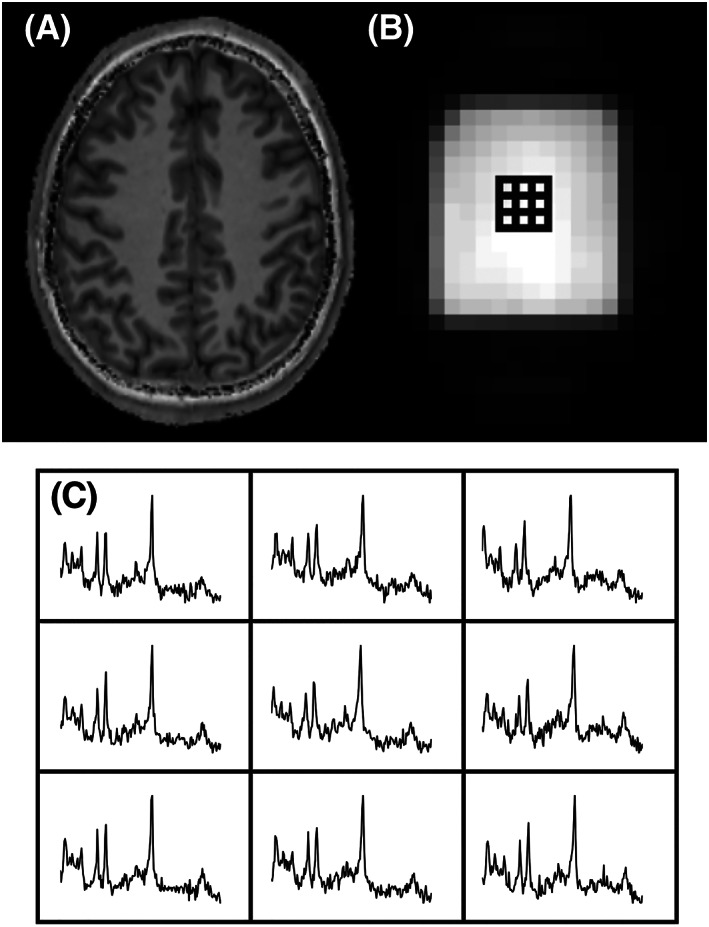
2D‐PEPSI in the human brain at 3 T using *T*_R_ 2 s, spatial matrix 32 × 32, scan time 64 s. The anatomical reference image (A) and an additional water image (B; from a separate non‐water‐suppressed acquisition) show the location of the multi‐voxel spectra (C)

The original EPSI technique, also known as symmetric EPSI or proton EPSI (PEPSI), uses a series of alternating positive and negative trapezoidal gradients to produce a zig‐zag trajectory in *k*_*x*_‐*t* space.^3^ Ideally, the acquired *k*_*x*_‐*t* data can be sorted into a matrix after phase correction of the negative echo data and reconstructed using Fourier transform. In practice, the positive and negative echo data are not equivalent due to asymmetries in gradient switching and eddy currents, and direct Fourier transform reconstruction of combined positive and negative echoes would lead to ghosting artifacts in the spectral domain. One way to avoid spectral ghosting is to perform a separate Fourier transform for positive and negative echoes followed by combination after phase correction, at the expense of halving the SBW.^75^ If data are acquired during the gradient ramps, there is only a small penalty in SNR/*t* compared with phase‐encoded MRSI, despite the significant acceleration (e.g., 32‐fold).^73^, ^74^ Figure 6 shows PEPSI results in the human brain. Alternatively, shift correction between positive and negative echo data can be performed using an interlaced Fourier transform approach to exploit the full SBW.^97^ Center‐out EPSI readout, where the upper half of *k*‐space is acquired during the first segment and the lower half of *k*‐space during the second segment, has also been proposed to passively prevent formation of ghosting artifacts and optimize SBW, by computing the shift correction between positive and negative echo data directly from the differences between upper‐half and lower‐half *k*‐space data.^98^

EPSI implementations have been shown for various pre‐localization schemes including PRESS,^79^ semi‐LASER (localization by adiabatic selective refocusing),^78^whole slice with OVS^99^ and slab‐selection.^100^

MRSI techniques that require multiple repetitions with different parameter settings benefit particularly from EPSI. This includes diffusion‐weighted MRSI, the first implementation of which was shown in 1995,^101^ followed by more robust versions recently,^102^ with some even enabling diffusion tensor imaging of metabolites.^103^ Combining MRSI with encoding of two frequency dimensions simultaneously (e.g., *J*‐resolved or correlation spectroscopy) is even more time consuming and has therefore been an active field of research. Correlated MRS (COSY)‐EPSI has been predominantly used to map muscle metabolism,^104^, ^105^, ^106^, ^107^, ^108^ while *J*‐resolved EPSI has been applied to prostate^109^ and investigations in the human brain.^110^, ^111^, ^112^, ^113^, ^114^ Spectrally edited MRSI requires subtraction of one spectrum from another, which doubles the scan time and prohibits short *T*_R_, and hence can be efficiently accelerated by EPSI.^115^ Other less common applications include rapid temperature^116^ and metabolite *T*_2_ mapping.^117^

Unquestionably, the most common application of EPSI is to enable time‐efficient whole‐brain MRSI,^100^ which has reached a high level of sophistication and automation after many optimization steps.^118^, ^119^ It is used in clinical investigations for various brain disorders such as brain tumors,^120^, ^121^, ^122^, ^123^ amyotrophic lateral sclerosis,^124^ schizophrenia^125^ or dyslexia.^126^ Applications outside the brain, including MRSI of the breast,^127^, ^128^ liver^129^ and calf muscle,^130^ have been proposed as well.

#### 3.1.2 Flyback‐EPSI and more

To reduce SBW limitations, the flyback‐EPSI technique uses only the positive gradient part for spatial encoding and short gradient pulses with maximum slew rate for refocusing. The flyback‐EPSI readout also mitigates eddy current effects and ghosting significantly, but at the loss of SNR due to gaps in data acquisition.^131^ Other less common approaches to increase the SBW are temporal interleaving and repeating the acquisition with reversed readout gradients, which double both the SBW and the scan time.^79^, ^132^ Another solution that doubles the SBW of EPSI without prolonging the scan time is coherent *k*‐*t* space EPSI.^82^, ^133^ Finally, multi‐shot EPSI was proposed, which samples not only a single *k*‐space line along *k*_*x*_, but a complete echo‐planar trajectory to cover a large fraction of the *k*_*x*_‐*k*_*y*_ plane with gaps in *k*_*y*_ and time being filled up by the following shots, but this caused significant spectral aliasing artifacts and imposes SBW limitations.^134^ Flyback‐EPSI has been applied clinically in brain tumors,^135^, ^136^ prostate cancer^137^ and multi‐nuclear MRSI (i.e., hyperpolarized ^13^C‐MRSI^138^ and ^31^P‐MRSI^139^ of the calf muscle).

### 3.2 Non‐Cartesian SSE

Non‐Cartesian SSE techniques accelerate in two *k*‐space dimensions and the spectral dimension simultaneously with only a few exceptions.^140^ The most prominent examples are spirals,^76^ concentric ring trajectories (CRTs),^80^ rosettes^81^ and radial EPSI.^96^ The most common reconstruction approach for non‐Cartesian trajectories is to perform a non‐uniform fast Fourier transform (NUFFT) in the spatial domain and conventional fast Fourier transform (FFT) along the temporal domain.

#### 3.2.1 Spirals

**FIGURE 7:**
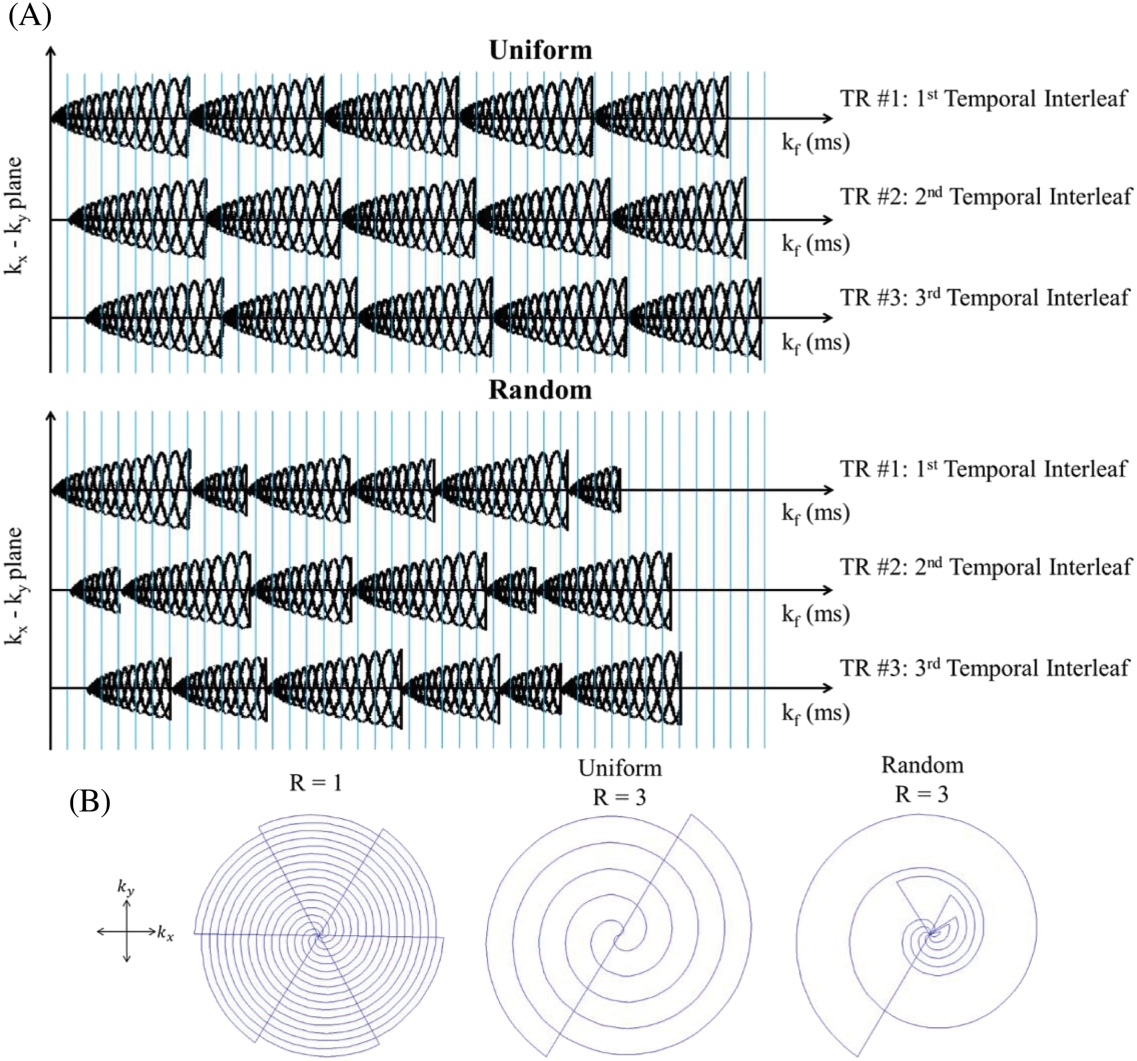
a: Two types of spiral‐based *k*‐space acquisition schemes illustrate the concept of spectral interleaves: Uniformly (top) and randomly (bottom) undersampled spiral *k*‐space acquisition schemes. The blue vertical lines represent the time at which a sample is acquired. b: The projections of possible *k*‐space trajectories onto the k_x_‐k_y_ plane illustrate the concept of spatial (angular) interleaves. The projection from fully sampled (all six spatial interleaves), uniformly undersampled (*R* = 3, only two of six spatial interleaves), and randomly undersampled (R = 3) acquisition are shown from left to right, respectively. Reproduced from Chatnuntawech et al^142^

Historically, the first non‐Cartesian SSE *k*‐space trajectory was the spiral.^76^ This was motivated by the early application of spirals in MRI and the fact that nominally constant‐density spirals are the most efficient trajectories, offering the best acquisition time and SBW benefit while sacrificing the least SNR, but they are also more susceptible to gradient infidelities than other SSE techniques,^86^ which requires corrections.^67^ For small matrix sizes and lower SBW (e.g at 1.5 T), spirals can acquire spectroscopic data in a single shot, but single‐shot approaches are usually not practical due to SBW limitations imposed by the time required to complete the spiral trajectory. In practice, spiral MRSI is, therefore achieved via spectral (Figure 7A) and spatial interleaves (Figure 7B) (i.e., acquiring only a fraction of the *k*‐space or FID, respectively, per *T*_R_).^141^

With these properties, spirals are ideal for application in hyperpolarized ^13^C studies, where speed is critical to minimize *T*_1_ relaxation‐related SNR losses,^37^, ^143^, ^144^, ^145^, ^146^, ^147^ but also more recently in dynamic ^31^P‐MRSI in muscles,^30^, ^148^ where a high temporal resolution is essential. Spiral SSE is also playing an increasingly important role in ^1^H‐MRSI of the brain^71^, ^149^, ^150^, ^151^, ^152^, ^153^, ^154^, ^155^, ^156^, ^157^, ^158^ and prostate^141^, ^159^, ^160^ to reach clinically attractive scan times. At 3 T fully scanner‐integrated solutions for spiral MRSI^154^, ^156^ have recently facilitated a stronger clinical use for brain tumor,^150^, ^152^, ^153^ neurodegenerative^151^, ^161^ and demyelinating disorders,^161^ as well as psychiatric research.^149^, ^163^

Since the evolution of ^1^H‐MRSI towards whole‐brain coverage^76^, ^164^ there is an increased need for mitigation of extra‐cranial lipid bleeding artifacts. This has led to the development of variable‐density spirals, which improve the SRF during the acquisition in an SNR‐efficient manner without the need for inefficient post‐processing *k*‐space filters.^142^, ^165^, ^166^, ^167^, ^168^ This is frequently augmented by preprocessing to remove (lipid) artifacts even further.^166^, ^167^, ^168^, ^169^, ^170^

However, due to limitations imposed by common whole‐body gradients the efficiency of spirals suffers with increasing spatial resolution and SBW, which is typical for high‐field MRSI.^5^, ^41^ For SSE the same trajectory must be repeated several hundred times to sample an FID, but gradient slew rate limitations do not allow return to the *k*‐space center after each spiral sufficiently fast. All data acquired during such gradient rewinders (i.e., similar to flyback‐EPSI gradients) are unused, which lowers the SNR.^16^ This makes “closed loop”‐trajectories without such a deadtime attractive for SSE. In‐out spirals feature such self‐rewinding properties, but they are only efficient for strong animal gradient systems,^171^, ^172^ not for whole‐body systems, where additional limitations on maximum SBW would be imposed.

#### 3.2.2 Concentric rings

**FIGURE 8:**
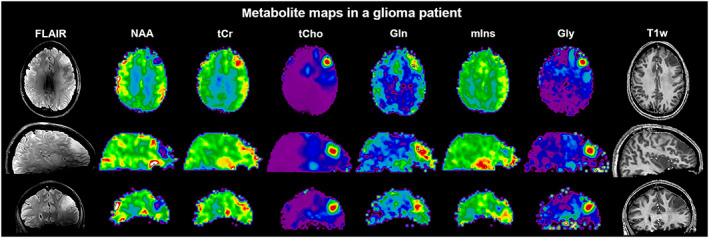
Sample maps of six major metabolites obtained in a brain tumor patient (30‐year‐old female; anaplastic astrocytoma grade 3 with suspected progression into glioblastoma) along with conventional *T*_1_‐ and *T*_2_‐weighted MRI displayed in transversal, sagittal and coronal planes. Whole‐brain FID‐MRSI data were acquired at 7 T in 15 min using a 3D CRT sequence with variable temporal interleaves, *T*_R_ 450 ms, acquisition delay 1.3 ms, spatial resolution 3.4 × 3.4 × 3.4 mm^3^, 64 × 64 × 39 matrix and SBW 2778 Hz. The *T*_2_‐weighted FLAIR image is strongly affected by *B*_1_ inhomogeneities, while this is less of a problem for gradient‐echo‐based images such as the *T*_1_‐weighted MRI and metabolic maps. Courtesy of Gilbert Hangel

These problems have triggered the development of inherently closed SSE trajectories without deadtimes, which are not merely translations of existing MRI trajectories, but rather tailored for the needs of MRSI. CRTs are the best example of this. They were originally proposed for hyperpolarized ^13^C‐MRSI,^80^, ^86^ but were rapidly adapted for ^1^H‐MRSI with high spatial resolution and SBW.^7^, ^63^, ^64^, ^173^, ^174^, ^175^, ^176^ Via CRTs an *N* × *N* matrix resulting from a *k*_*N*_ × *k*_*N*_ *k*‐space is covered by *k*_*N*_/2 equidistant rings, which makes CRT‐based SSE exactly twice as fast as EPSI (i.e., it requires *k*_*N*_ lines). For equidistant CRTs the acquired *k*‐space is inherently 1/*k*_r_ weighted, but the weighting can be optimized (e.g Hanning weighting) at the expense of reduced acceleration.^63^, ^173^ The constant‐angular‐velocity properties of CRTs make them robust to gradient timing imperfections and eddy current delays,^86^ and PI reconstruction is considerably simplified.^64^ A unique feature of CRTs is that scan time/SNR efficiency can be further improved by acquiring a variable number of temporal interleaves (one temporal interleave/circumnavigation is sufficient in the *k*‐space center, while in the *k*‐space periphery gradient slew rate limits demand two or three temporal interleaves).^7^ This makes high‐resolution whole‐brain ^1^H‐MRSI clinically feasible even at UHF (Figure 8).

#### 3.2.3 Rosettes

Rosettes are the other class of closed non‐Cartesian SSE trajectories.^81^, ^177^, ^178^, ^179^ A main feature of rosettes is their design flexibility, which allows tailoring of the trajectories for desirable features such as speed, low‐gradient performance, repeated sampling of the *k*‐space center or adapting the *k*‐space weighting. Depending on the parameter settings, rosettes can become identical to rings, in‐out spirals or radial EPSI.^179^ The ability to tailor rosette trajectories for low‐gradient performance and reduced acoustic noise could make them useful in the regime of very high SBW/spatial resolution. MRI results indicate that rosettes can be more incoherently undersampled than other non‐Cartesian trajectories, which may be a benefit for CS reconstruction.^180^

#### 3.2.4 Radial EPSI

Finally, MRSI based on radial EPSI (again originally proposed for hyperpolarized ^13^C‐MRSI^96^) is a fairly young field. Only recently have preliminary experimental reports in ^1^H‐MRSI and ^31^P‐MRSI been published.^181^, ^182^ Like spirals, radial EPSI trajectories return to the *k*‐space center, which offers in principle the possibility for self‐navigation (i.e., correction by phase/magnitude alignment of consecutive trajectories). This can be used to reduce instabilities (e.g., motion) between different *k*‐space interleaves (Figure S2),^182^, ^183^ while other SSE techniques (e.g., EPSI) require interleaved navigators for this.^184^

## 4 UNDERSAMPLED MRSI

**FIGURE 9:**
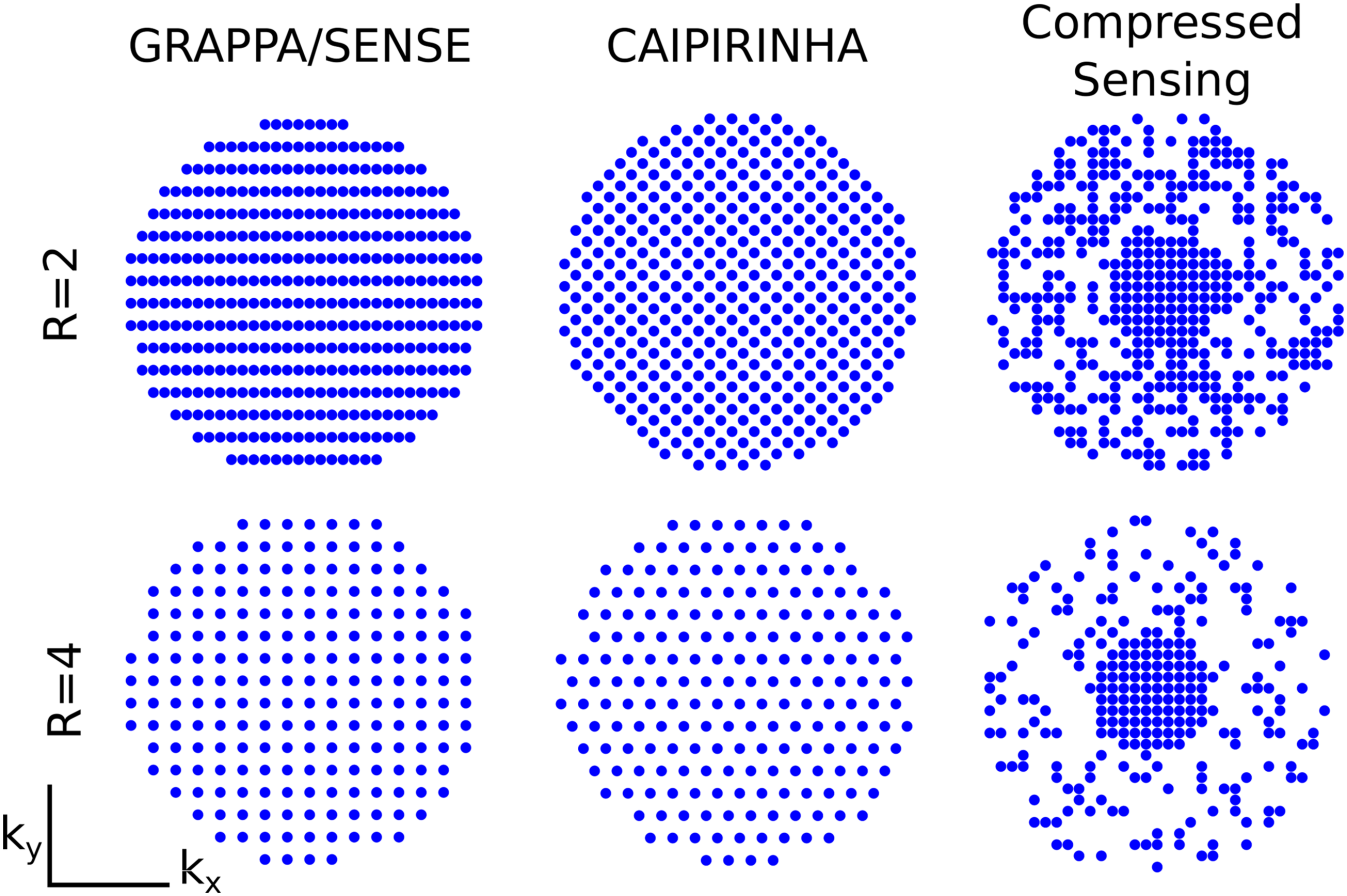
Illustration of different 2D *k*‐space undersampling schemas for two‐fold acceleration (*R* = 2) and four‐fold acceleration (*R* = 4). GRAPPA/SENSE can reconstruct coherently sampled *k*‐space data (e.g., entire rows or columns are not acquired), while CAIPIRINHA can reconstruct even coherently undersampled data with any other patter and benefits from controlled aliasing. All of them use information about sensitivity profiles of the individual receive channels to remove spatial aliasing either in the image (e.g., SENSE) or *k*‐space domain (e.g ., GRAPPA, CAIPIRINHA). This translates into better reconstruction with lower *g*‐factors (e.g ., less lipid aliasing and higher SNR). In contrast, CS can reconstruct incoherently (e.g., random‐like) undersampled *k*‐space data without knowledge about the coil receive profiles of the individual receive channels. Courtesy of Lukas Hingerl

*k*‐space undersampling is the third major acceleration method for MRSI. For an unambiguous signal allocation by gradient encoding, the distance between all adjacent *k*‐space points must be less than 1/object size (i.e., Nyquist criterion). Conventional phase‐encoded MRSI at the Nyquist rate (i.e., full *k*‐space sampling) is very time consuming. In undersampled MRSI, fewer *k*‐space points are acquired below the Nyquist rate to speed up the acquisition using coherent and incoherent undersampling patterns (Figure 9). Complementary information other than gradient encoding, which includes sensitivity maps, spatial‐spectral sparsity or prior knowledge, is employed to reconstruct the undersampled data sets without aliasing.

### 4.1 Parallel imaging

A common group of methods for reconstruction of k‐space undersampled MRI data is PI.^185^, ^186^, ^187^ In classic implementations, *k*‐space undersampling is performed in a uniformly equidistant manner across *k*‐space (i.e., regular pattern), yielding a larger distance between sampled *k*‐space points. This in turn leads to a field‐of‐view reduction to below the dimensions of the object and thus to aliasing (folding) of image information. To reconstruct the missing *k*‐space points or to unfold the aliased image additional information on the spatial signal origin is derived from sensitivity profiles of receive coil arrays. There are two major reconstruction approaches in PI that have found widespread application: (i) sensitivity encoding (SENSE)^187^ and (ii) generalized autocalibrating partially parallel acquisition (GRAPPA).^186^ SENSE solves the image reconstruction problem in the image domain by unfolding the aliased images, and requires explicit sensitivity maps of each coil element to form an overdetermined system of linear equations. Both the *k*‐space encoding trajectory and receiver coil sensitivity patterns are input to the algorithm. GRAPPA solves the same problem in *k*‐space domain, and typically only undersamples the outer parts of the *k*‐space to derive a reconstruction kernel that predicts the missing *k*‐space points from the central fully sampled part of *k*‐space. Both principles are widely applied in MRI and have been demonstrated for ^1^H‐MRSI as well. PI is generally also compatible with SSE and incoherent *k*‐space undersampling (see Section 5).

#### 4.1.1 SENSE

**FIGURE 10:**
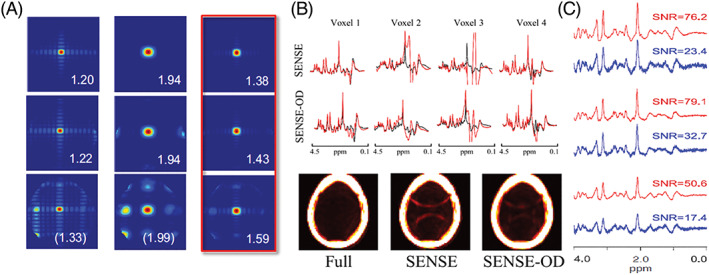
Lipid aliasing control by overdiscrete SENSE MRSI reconstruction. A, SRFs for different acceleration factors (top row ‐ R=1; middle row ‐ R=2; bottom row ‐ R=2x2) after standard SENSE reconstruction (left column), a Hamming filtered version of it (middle column) and an overdiscrete, spatial‐response function target based SENSE reconstruction (right column). B, Spectra and lipid maps from fully sampled, SENSE and SENSE‐OD reconstruction (2 × 2 acceleration each) of ^1^H‐FID‐MRSI data from 9.4 T. C, SNR after SENSE (blue) and SENSE‐OD (red) reconstruction of 7 T ^1^H‐FID‐MRSI data. Reproduced from the work of Kirchner et al.^192^, ^193^ and Nassirpour et al^55^

Sensitivity‐encoded ^1^H‐MRSI was introduced shortly after the invention of SENSE‐MRI in 2001 and was combined with different localization schemes such as PRESS,^188^ slice‐selective adiabatic SE localization,^189^ SE‐ or FID‐MRSI with OVS^190^, ^191^, ^192^, ^193^ or FID‐MRSI without OVS.^55^ This pre‐localization was necessary because the original SENSE‐MRSI implementation suffered from residual lipid aliasing artifacts introduced by imperfections of the sensitivity maps and insufficient control of the SRF. The ESPIRIT approach^55^, ^192^, ^194^, ^195^ to derive reliable sensitivity maps enhanced the robustness and applicability of SENSE‐MRSI since it is free of residual bias fields or image contrasts, is compatible with transceiver arrays and is free of interpolation errors towards the edge of the object of interest. Several methods to further reduce lipid aliasing were presented, and include direct optimization of the SRF, overdiscrete or superresolution reconstruction and retrospective lipid removal.^55^, ^193^, ^196^, ^197^, ^198^ SENSE was also utilized for an overdiscrete *B*_0_‐correction that enhances the SNR of MRSI data.^55^, ^192^ Figure 10 illustrates controls of SRF, resulting lipid artifacts, and SNR increase. SENSE‐MRSI was combined with alternative acceleration methods such as elliptical *k*‐space sampling,^199^ multi‐echo MRSI,^20^, ^21^ EPSI,^190^, ^191^, ^200^ spiral MRSI,^142^ CS,^57^ low‐rank reconstruction^58^ and partial Fourier imaging.^201^ SENSE‐MRSI was also used to accelerate the acquisition of unsuppressed water maps for internal water referencing^202^ and a phantom‐based external referencing for quantitative SENSE‐MRSI compatible with receive arrays.^203^ SENSE‐MRSI was applied to clinical studies in brain tumor patients,^196^, ^204^ and shortly after its introduction SENSE‐MRSI was also implemented as a commercial option by a major vendor and is utilized in clinical diagnostics today on a regular basis. SENSE‐MRSI is not easily applicable to ^31^P‐, ^13^C‐ and ^2^H‐MRSI due to the lack of a high‐SNR reference standard (e.g., water in ^1^H‐MRI/MRSI) that allows for generation of high‐quality sensitivity maps. Nevertheless, applications of SENSE to hyperpolarized ^13^C‐MRSI have been demonstrated^201^, ^205^ sensitivity maps have been derived either by self‐calibration exploiting the high SNR of in vivo pyruvate and a fully sampled *k*‐space center^205^ or by using an oil phantom.^201^, ^206^

#### 4.1.2 GRAPPA

The earliest description of GRAPPA‐accelerated ^1^H‐MRSI stems from 2006.^207^ Implementations have been demonstrated that relied on PRESS pre‐localization,^204^, ^207^ slice‐selective or volumetric SE‐^65^, ^208^ and FID‐MRSI.^52^, ^56^, ^64^, ^209^ To reduce lipid aliasing in SE‐ or FID‐MRSI, lipid suppression by either OVS or double inversion recovery were presented,^190^, ^209^ but both options limit the acquisition speed by demanding higher *T*_R_ values due to SAR. Controlled aliasing in parallel imaging results in higher acceleration (CAIPIRINHA)‐MRSI encoding in combination with retrospective lipid removal yielded significantly better metabolite maps.^52^, ^167^ Finally, training neural networks for GRAPPA reconstruction on MRI data^56^ or acquiring the reference data for GRAPPA reconstruction interleaved^64^ reduced lipid aliasing to a negligible level. GRAPPA‐MRSI was combined with alternative acceleration methods such as elliptical *k*‐space sampling,^210^ EPSI,^65^, ^208^, ^211^ CRT,^64^ and spirals.^212^ Recently, through‐time/through‐*k*‐space GRAPPA was presented, which simultaneously yields an unsuppressed water reference scan.^64^ The impact of GRAPPA‐accelerated EPSI on diagnostic sensitivity in traumatic brain injury patients was investigated and identical metabolic changes were found.^211^ GRAPPA is generally compatible with non‐proton MRSI, but preliminary ^31^P‐MRSI implementations were limited by low SNR.^213^ A simultaneous auto‐calibrating and *k*‐space estimation (SAKE) variant was applied to hyperpolarized ^13^C‐MRSI.^195^, ^214^, ^215^

Early implementations of SENSE‐MRSI and GRAPPA‐MRSI have been compared, and favorable results have been obtained for conventional GRAPPA due to SNR advantages and lower lipid aliasing.^204^ SENSE‐MRSI was also compared against elliptical *k*‐space shuttering and EPSI, and it was concluded that all three methods are applicable to clinical diagnostics of brain tumors with individual advantages and disadvantages.^216^ However, the full potentials of neither SENSE nor GRAPPA have been exploited in these studies, and a more thorough comparison of state‐of‐the‐art SENSE and GRAPPA reconstruction algorithms and alternative sampling schemes has yet to be performed.

### 4.2 Multi‐band/simultaneous multi‐slice

**FIGURE 11:**
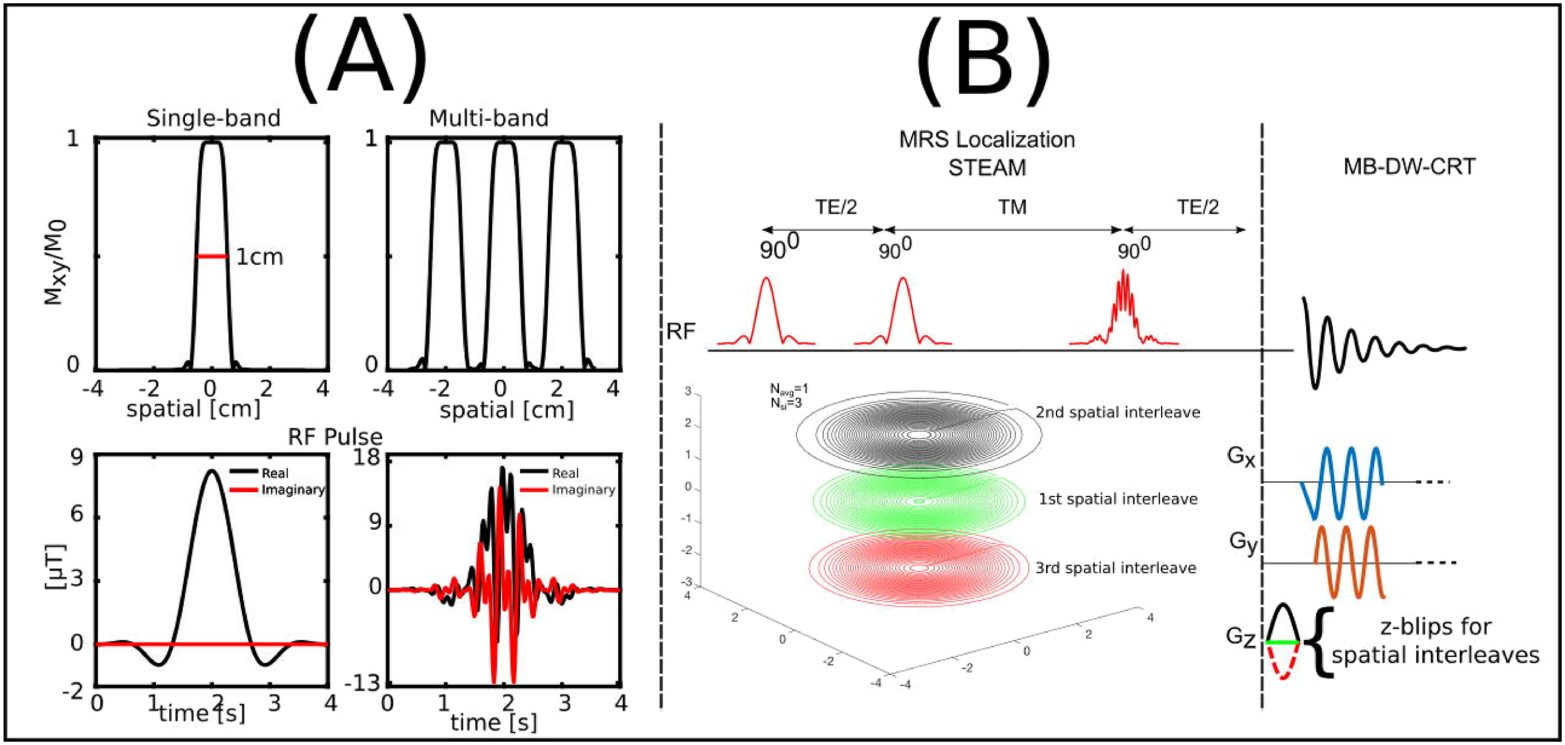
A, RF pulse and corresponding slice excitation profiles of single‐band and multi‐band pulses. The phase modulation of the multi‐band pulse reduced the peak *B*_1_^+^ while keeping the bandwidth of each band and pulse duration the same. B, The proposed multi‐band density‐weighted CRT MRSI method using the third STEAM pulse as a multi‐band excitation pulse. 96 rings with different radii are distributed evenly in three sets of rings with different *z*‐blip gradients. The single‐slice sequence has the same 96 rings in the *k*_*x*_‐*k*_*y*_ plane without encoding with *z*‐blip gradients. Reproduced from the work of Xia et al^220^

Another possibility to exploit the sensitivity profiles of multi‐channel receive coils for MRI acceleration is simultaneous multi‐slice (SMS) imaging.^217^ SMS makes efficient use of simultaneous excitation of several slices by one multi‐band RF pulse^218^ in combination with controlled aliasing^219^ and reconstructs the individual slices using the PI concept (Figure 11). The achievable acceleration factors are fairly low (~2 to 4) and can therefore only be used as an add‐on to either undersampling^52^ or SSE techniques,^82^, ^220^as shown for preliminary ^1^H‐MRSI^82^, ^220^ studies. The excitation of multiple slices via SMS should be not confused with multi‐band spectral‐spatial excitation (ie exciting different frequency bands simultaneously), which has been employed for rapid ^13^C‐MRSI.^38^, ^92^, ^221^, ^222^

### 4.3 Compressed sensing

CS‐MRI relies on a combination of non‐uniform *k*‐space undersampling and the assumption of spatial and/or spectral sparsity.^223^ Spatial or spectral sparsity means that there are relatively few significant voxels or spectral points with non‐zero values. During the image reconstruction, a wavelet and/or other compression transformation such as total variation or principal component analysis is performed to yield a sparse representation of the data similar to data compression in JPEG. *k*‐space undersampling must be incoherent to yield a noise‐like distribution of nuisance signals, which can be removed by a non‐linear reconstruction algorithm that enforces sparsity in the transformation domain (e.g., wavelet space) and is consistent with the acquired data.

The first implementation of CS‐MRSI was demonstrated in 2008 with application in hyperpolarized ^13^C‐MRSI,^36^ which still yields the majority of publications utilizing this MRSI acceleration method.^35^, ^36^, ^38^, ^85^, ^224^, ^225^, ^226^, ^227^, ^228^, ^229^, ^230^, ^231^ CS is ideal for hyperpolarized ^13^C‐MRSI due to its intrinsic spectral sparsity, which allows for additional acceleration along the spectral dimension. CS can be combined with SSE to further accelerate the metabolic imaging readout for hyperpolarized ^13^C‐MRSI to reach sufficient coverage and resolution in the presence of strict time limits,^35^, ^36^, ^85^, ^228^, ^231^ and was demonstrated for flyback‐EPSI, a custom designed incoherent spatial‐spectral undersampling scheme and EPI with frequency selective excitation. Other acceleration methods that have been combined with CS for hyperpolarized ^13^C‐MRSI are multi‐band encoding,^38^ multi‐point Dixon encoding^227^, ^230^ and balanced SSFP.^229^ Applications of CS in ^13^C‐hyperpolarization studies have focused mainly on cancer imaging.^35^, ^224^, ^225^, ^226^

Similarly, ^31^P‐MRSI is well suited for this acceleration approach due to well separated spectral lines and the absence of large nuisance signals. The first CS ^31^P‐MRSI implementation was shown in 2012 and was based on simulated 2D‐MRSI data.^232^ An actual 2D implementation for human brain ^31^P‐MRSI was shown in 2017.^148^, ^233^, ^234^ CS has been recently combined with flyback‐EPSI for highly accelerated ^31^P‐MRSI,^62^ and two distinct reconstruction schemes—L1‐norm minimization and low‐rank Hankel matrix completion—have been compared.^138^

**FIGURE 12:**
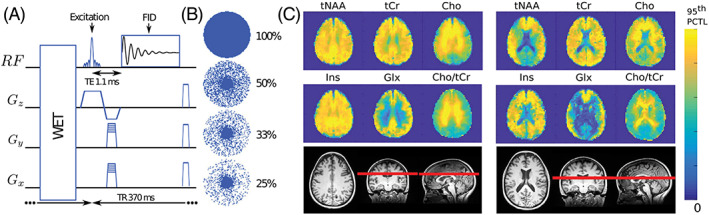
A, Diagram of the FID‐MRSI sequence with RF signal and gradient intensities along *z*, *y* and *x* directions (*G*_*z*_, *G*_*y*_, *G*_*x*_) and B, illustration of the elliptical incoherent *k*‐space sampling schemes corresponding to acceleration factors 1, 2, 3 and 4 . C, Metabolite distributions from two healthy volunteers reconstructed with a low‐rank Total Generalized Variation model with optimal regularization parameter (*λ* = 10^−3^) and rank *K* = 20 without acceleration. The maximum of the scale was set to the 95th percentile of each metabolite image separately. Reproduced from the work of Klauser et al^58^

The application of CS to ^1^H‐MRSI is complicated due to large water and lipid nuisance signals, which can mislead the reconstruction algorithm to exclude the lower‐intensity metabolite peaks by misadjusted thresholding. In addition, ^1^H spectra are not sparse (short‐*T*_E_ MRS in particular), making acceleration along the spectral dimension more challenging. The first CS ^1^H‐MRSI implementation was shown in 2009 in vitro^235^ and 2012 in vivo via retrospective undersampling.^236^ Several studies investigated the dependence of the SRF on the SNR and sampling pattern in CS‐accelerated ^1^H‐MRSI using phantom data.^237^, ^238^, ^239^ In vivo applications of CS ^1^H‐MRSI were combined with either PRESS^142^, ^236^, ^240^, ^241^ or semi‐LASER^85^ pre‐localization or slice‐selective ^1^H‐FID‐MRSI^57^, ^58^ (Figure 12). CS was used to further accelerate 3D *J*‐resolved EPSI for ^1^H‐MRSI prostate applications.^240^, ^241^ In addition the combination of CS with SENSE,^57^ CS with SENSE and incoherently undersampled spiral trajectories^142^ as well as CS and a low‐rank constrained reconstruction scheme have been demonstrated for ^1^H‐MRSI.^58^

## 5 JOINT FORCES—COMBINING SSE, UNDERSAMPLING AND SHORT *T*_R_

Even though SSE by itself can provide up to two magnitudes of acceleration compared with pure phase encoding, this is often not sufficient for reaching the desired target temporal resolution^38^, ^200^ or volume coverage (e.g., whole brain) with sufficiently high spatial resolution.^64^, ^208^, ^211^ In such cases, SSE techniques can be combined with PI,^64^, ^80^, ^190^, ^208^, ^211^ CS^35^, ^36^, ^38^, ^228^ or a mixture of the two techniques.^142^ Such undersampling can be performed in spatial (*k*_*x*_, *k*_*y*_), spectral (*t*) and dynamic (frame) dimensions. The possibility to undersample in multiple dimensions in an entangled fashion (e.g., both *k*‐space and time domain) is highly beneficial for reconstruction efficiency.^242^ The high sparsity in the spectral dimension is particularly appealing for CS and can be efficiently exploited in conjunction with SSE by using a different *k*‐space undersampling for each FID point to introduce spectral incoherence (e.g., by adding random phase‐encoding blips).^142^

### 5.1 Cartesian

EPSI techniques simultaneously encode one spatial dimension and the spectral dimension, but the other spatial dimensions are still acquired using conventional phase encoding, and thus the scan time increases proportionally. PI can be used to regularly undersample the phase‐encoding dimensions of EPSI and exploit differences in coil sensitivities to reconstruct unaliased spectroscopic images. 1D‐SENSE^191^ and 1D‐GRAPPA^65^ were employed to accelerate 2D‐EPSI in the brain twofold for an 8‐channel coil array and threefold for a 32‐channel array, resulting in acquisition times below 1 min for a 32 × 32 spatial matrix and *T*_R_ of 2 s. Acceleration in PI is limited by noise amplification due to a reduced number of phase‐encoding points and instability in the inverse reconstruction produced by overlapping coil sensitivities (the so‐called *g*‐factor). Higher accelerations are feasible with the use of 2D undersampling for 3D imaging and a coil array with a large number of elements to enable 2D‐SENSE, which reduce the *g*‐factor. For example, 2D‐SENSE using a 32‐channel soccer ball head coil array enabled an acceleration factor of 2 × 2 for 3D‐PEPSI with no additional degradation in spatial‐spectral quality beyond the expected SNR decrease of sqrt(*R*), resulting in acquisition times of 2 min for a 32 × 32 × 8 spatial matrix (Figure S3).^190^

**FIGURE 13:**
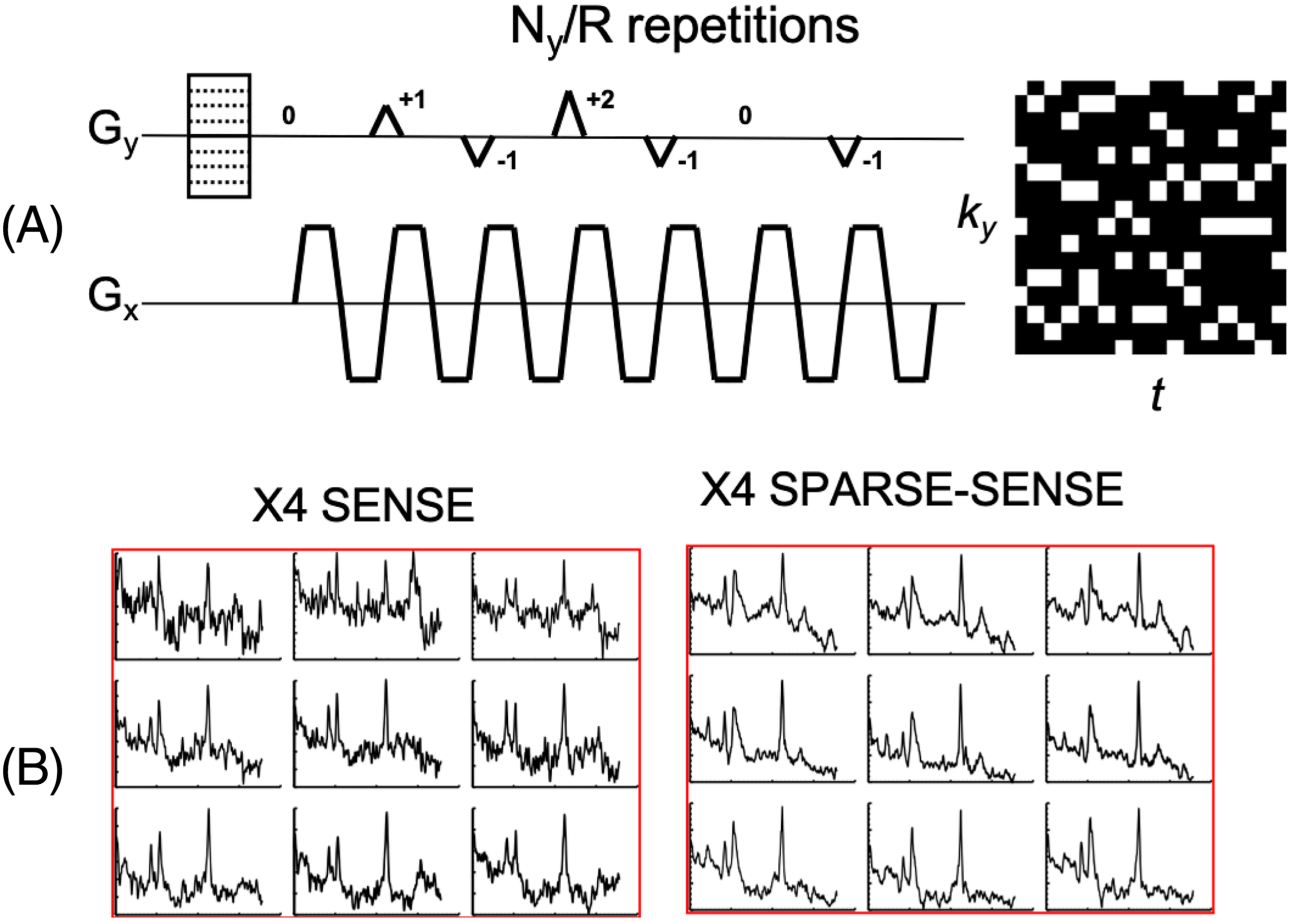
A, CS acquisition scheme for 2D‐PEPSI, based on random *k*_*y*_‐*t* undersampling using phase‐encoding blips. Each time point is undersampled with a different random *k*_*y*_ pattern to achieve spatial‐spectral incoherence. B, Comparison of real spectra obtained with fourfold acceleration for 2D‐EPSI using SENSE (PI) and SPARSE‐SENSE (combination of CS and PI). SPARSE‐SENSE jointly exploits sparsity in the spectral wavelet domain and coil SENSE by enforcing joint multi‐coil sparsity in the reconstruction to achieve significantly higher spectral quality and noise reduction compared with standard SENSE due to regularization. Reproduced from the work of Otazo et al^243^

An alternative approach to undersample the phase‐encoding dimensions of EPSI is CS, which can exploit the natural sparsity along the spectral dimension in long‐*T*_E_ acquisitions or transform sparsity for short‐*T*_E_ acquisitions. The application of typical sparsifying transforms, such as wavelets and principal component analysis, along the spectral dimension can lead to combined spatial‐spectral sparse representations of short‐*T*_E_ data. For 2D‐EPSI, spatial‐spectral incoherent sampling can be achieved by using phase‐encoding blips, which result in a different *k*_*y*_‐undersampling pattern for each time point^95^ (Figure 13). To increase the acceleration rate, CS can be combined with PI to enforce joint coil sensitivity sparsity in the reconstruction. SPARSE‐SENSE, which combines CS and SENSE, was applied to 2D‐PEPSI to achieve fourfold acceleration with significant improvements compared with standard SENSE.^243^ CS was also applied to *T*_E_‐averaged EPSI of glutamate to compensate for the increased scan time required to acquire at multiple *T*_E_ values.^244^ CS is also a natural candidate for acceleration of *J*‐resolved MRSI, given the substantial correlations in the high‐dimensional data. CS with fourfold acceleration was demonstrated for 4D echo‐planar correlated spectroscopic imaging to reduce the scan time to 5 min.^245^

### 5.2 Non‐Cartesian

**FIGURE 14:**
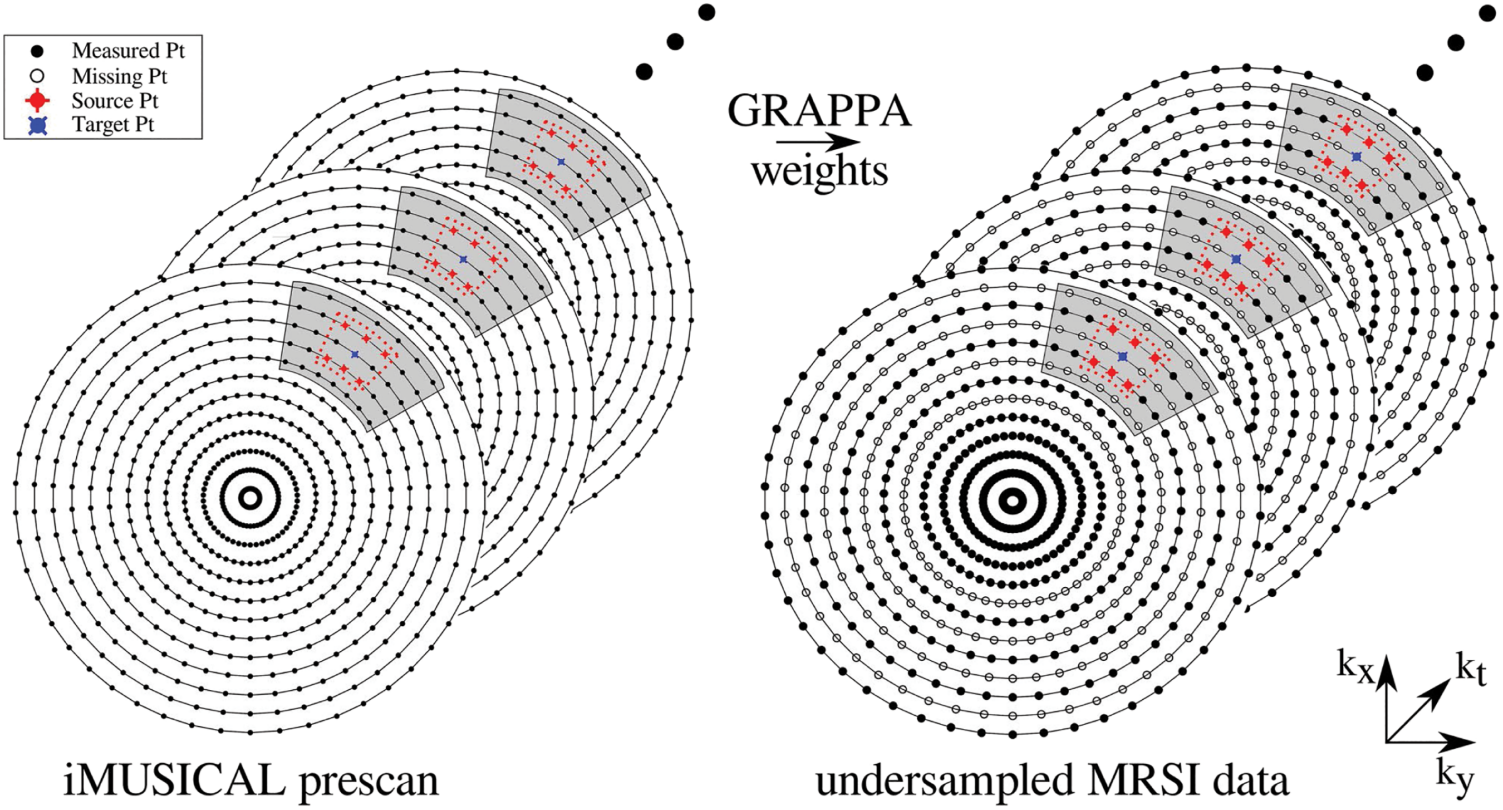
A schematic diagram of the through‐time/through‐*k*‐space GRAPPA method applied to concentric‐ring MRSI. The figure shows a fully sampled calibration dataset on the left and a two‐fold variable‐density undersampled MRSI *k*‐space on the right: the center of the *k*‐space is fully sampled (solid points), followed by constant two‐fold undersampling (empty points). For calibration, a segment (shaded area) was used around each target point for the through‐*k*‐space GRAPPA part with a kernel size of 3 × 2 (red dotted lines), i.e., six source points. The kernel was slid through the segment of the calibration data to gather all the through‐*k*‐space kernel repetitions. Twenty‐one calibration time points (through‐time kernel repetitions) were used. After calculating the GRAPPA weights using the calibration data, the weights are used to reconstruct missing *k*‐space points in the undersampled MRSI data (right). Reproduced from the work of Moser et al^64^

It is well known from MRI that non‐Cartesian trajectories, such as those also used for radial EPSI, spirals and CRTs, can be more efficiently accelerated via PI than Cartesian trajectories, meaning that the *g*‐factor‐related SNR penalty is lower.^246^ This is a consequence of the less structured aliasing artifacts that are obtained via non‐Cartesian sampling, which translates into more efficient reconstruction compared with Cartesian counterparts.^242^ This has also been suggested experimentally for MRSI using spirals^212^, ^247^ and CRTs (non‐Cartesian *k*‐space undersampling and reconstruction approaches illustrated in Figure 14).^64^, ^80^ SMS, which exploits sensitivity profiles as well, can therefore also be more efficiently combined with non‐Cartesian SSE (e.g., CRTs).^220^ Additionally, CS reconstruction benefits from variable‐density *k*‐space sampling,^223^ which can be easily achieved via non‐Cartesian spatial encoding.

Despite this evidence, the number of reports that have combined non‐Cartesian SSE with undersampling is still limited compared with Cartesian SSE. This is partially related to the more complicated and often time‐consuming reconstructions (e.g., iterative).^246^, ^248^ With its excess SNR, hyperpolarized MRSI is a prime candidate to benefit from undersampled non‐Cartesian MRSI. On the other hand, there is a lack of good multi‐channel multi‐nuclear coils that would be necessary to perform PI efficiently for hyperpolarized ^13^C‐MRSI, leaving CS—in particular in the spectral domain—the only option for further acceleration. In fact, spectral aliasing due to undersampling may not necessarily have to be undone for hyperpolarized ^13^C‐MRSI as long as it does not cause major spectral overlap of important metabolite resonances.^249^ In this context, it should be noted that spectral aliasing due to undersampling is more problematic for ^1^H‐MRSI data. Short‐*T*_E_ MR spectra are far less sparse (i.e., many strongly overlapping resonances) and there can be strong contamination by large nuisance signals (i.e., unsuppressed water or extra‐cranial lipids). This is not a problem for ^13^C‐ or ^31^P‐MRSI.^148^, ^234^ Nevertheless, highly efficient combination of variable density spirals and an entangled SENSE and CS reconstruction has so far been shown only for ^1^H‐MRSI at 3 T using PRESS.^142^ However, with an increasing number of better rapid non‐Cartesian reconstruction algorithms emerging for MRI,^250^ the interest in undersampled non‐Cartesian SSE for ^1^H‐MRSI is increasing.

## 6 USING PRIOR KNOWLEDGE

A promising approach to improve the results of highly accelerated MRSI is the incorporation of spatial (e.g., from high‐resolution anatomical MRI) and spectral (e.g., spectral components and their relaxation times) prior knowledge as well as bias field maps (*B*_0_ maps, *B*_1_^+^ maps) to reconstruct data with incoherent *k*‐space undersampling. More recently, low‐rank reconstruction schemes were combined with corresponding prior knowledge. The following paragraphs review the evolution of acquisition and reconstruction approaches based on prior knowledge.

### 6.1 Spatial prior knowledge and bias field maps: SLIM, SLOOP and SLAM

The utilization of prior knowledge derived from high‐resolution anatomical images for reconstruction of MRSI data with substantial *k*‐space undersampling was suggested as early as 1988, when the concept of spectral localization by imaging (SLIM)^251^ was introduced by Hu et al. The basic idea behind SLIM is to use a structural image to identify several compartments, each of which is assumed to be spatially uniform with regard to its metabolism. Hence, SLIM was originally used to derive spectra from a few arbitrarily shaped spatial compartments. For instance, a ^1^H‐MR image of a limb may show three regions containing muscle, fat and bone marrow.^251^ In principle, only three phase encoding steps are required to reconstruct three spectra from these three compartments. Applications of SLIM were reported for ^1^H and ^31^P‐MRS of tissue samples,^252^ perfused organs^253^ and in vivo skeletal muscle.^251^, ^254^ In 1991 spectral localization with optimal pointspread function (SLOOP) as an improvement to SLIM was suggested, which minimizes contaminations from other compartments by optimizing the *k*‐space sampling scheme,^255^ SLOOP was developed further to include additional prior knowledge on *B*_1_^+^, *T*_1_ and sequence parameters to optimize the SNR for human application of 3D‐encoded ^31^P‐MRS in the human myocardium.^256^ A further development step was the utilization of additional non‐linear gradients for the encoding of the SLOOP compartments.^257^ In vivo ^31^P‐SLOOP was mainly performed to characterize human cardiac muscle metabolism.^257^, ^258^, ^259^, ^260^, ^261^, ^262^, ^263^

SLIM developed into a metabolic imaging method, with the first report being the generalized series approach to MR spectroscopic imaging (GSLIM).^264^, ^265^ GSLIM allows for spatial variations inside the compartments (e.g., metabolite concentrations and *B*_0_ inhomogeneity), which are estimated from the data itself; the ill‐posedness of the problem is dealt with by using regularization techniques. GSLIM and natural linewidth chemical shift imaging (NL‐CSI) compensate for the sensitivity of SLIM to *B*_0_ inhomogeneity across the predefined compartments by incorporating prior knowledge from additional *B*_0_ maps into the reconstruction.^266^, ^267^ Static and radiofrequency‐compensated SLIM (STARSLIM) even incorporates prior knowledge about *B*_0_ and *B*_1_^+^ inhomogeneity.^268^ *B*_0_‐adjusted and sensitivity‐encoded spectral localization by imaging (BASE‐SLIM) incorporated *B*_0_ and sensitivity maps into the SLIM reconstruction.^269^ Finally, spectroscopy with linear algebraic modeling (SLAM)^270^, ^271^ was introduced, which substitutes the compartments in SLIM by a set of coalesced MRSI voxels with the same concentrations. Similar to SLOOP, SLAM chooses an optimized set of the same number of low‐gradient *k*‐space vectors as final compartments to maximize SNR and to minimize signal bleeding. SLAM has been combined with SENSE. Initial applications are ^31^P‐MRS in human skeletal muscle and myocardium^271^ and hyperpolarized ^13^C‐MRSI.^272^ A recent review paper gives a more comprehensive overview of all derivatives of SLIM and its applications in brain MRSI.^273^

An alternative reconstruction approach unrelated to SLIM incorporates *B*_0_ inhomogeneity as an additional encoding process together with the use of anatomical prior knowledge in a regularization term^274^ to improve MRSI reconstruction of undersampled data. Finally, the consideration of image prior knowledge as a direct constraint of the SRF along with *B*_0_ inhomogeneity correction has also been demonstrated for SENSE‐MRSI to better control lipid artifacts.^275^

### 6.2 Spatial‐spectral prior knowledge and low‐rank reconstruction: toward SPICE

In addition to spatial prior knowledge, spectral prior knowledge has been used to reconstruct accelerated MRSI data. The first reported use of spectral priors in MRSI reconstruction relates to *B*_0_ inhomogeneity correction by reference deconvolution using a spectral line shape model reflecting the *B*_0_ inhomogeneity distribution and aims at the improvement of the spectral linewidth.^276^, ^277^, ^278^ Early spatial‐spectral modeling in MRSI reconstruction combined the conventional gradient encoding data consistency term with additional regularization terms considering a combined spectral and baseline model term as well as a total variation term^279^, ^280^ and considered *B*_0_ inhomogeneity as an encoding mechanism.^281^ Another spatial‐spectral reconstruction approach aimed at controlling lipid spread and combined the usual consistency term that minimizes the difference between the gradient encoding data model and the data with a regularization or penalty term that includes a spatial‐spectral lipid model.^167^

Another important development step was the use of low‐rank approximation approaches in MRSI reconstruction,^58^, ^168^, ^282^, ^283^, ^284^, ^285^ the main idea of which is to remove basis vectors related to noise to yield higher‐SNR spectroscopic images. Following an initial application in denoising of MRSI data,^284^ low‐rank filtering was integrated with *B*_0_ inhomogeneity correction^283^ and additional image prior knowledge on tissue type boundaries^282^ for a more robust reconstruction of fully sampled Cartesian MRSI. Shortly afterwards, low‐rank approximation was combined with balanced‐SSFP or dynamic spiral hyperpolarized ^13^C‐MRSI with incoherent *k*‐space sampling^285^, ^286^ and ^1^H‐FID‐MRSI accelerated by short *T*_R_ and a combined SENSE and CS acceleration.^58^ Low‐rank reconstruction was also used together with a spatiotemporal lipid prior that assumes orthogonality of spatiotemporal metabolite versus lipid signals and applied to lipid‐unsuppressed dual‐density spiral ^1^H‐MRSI.^168^

**FIGURE 15:**
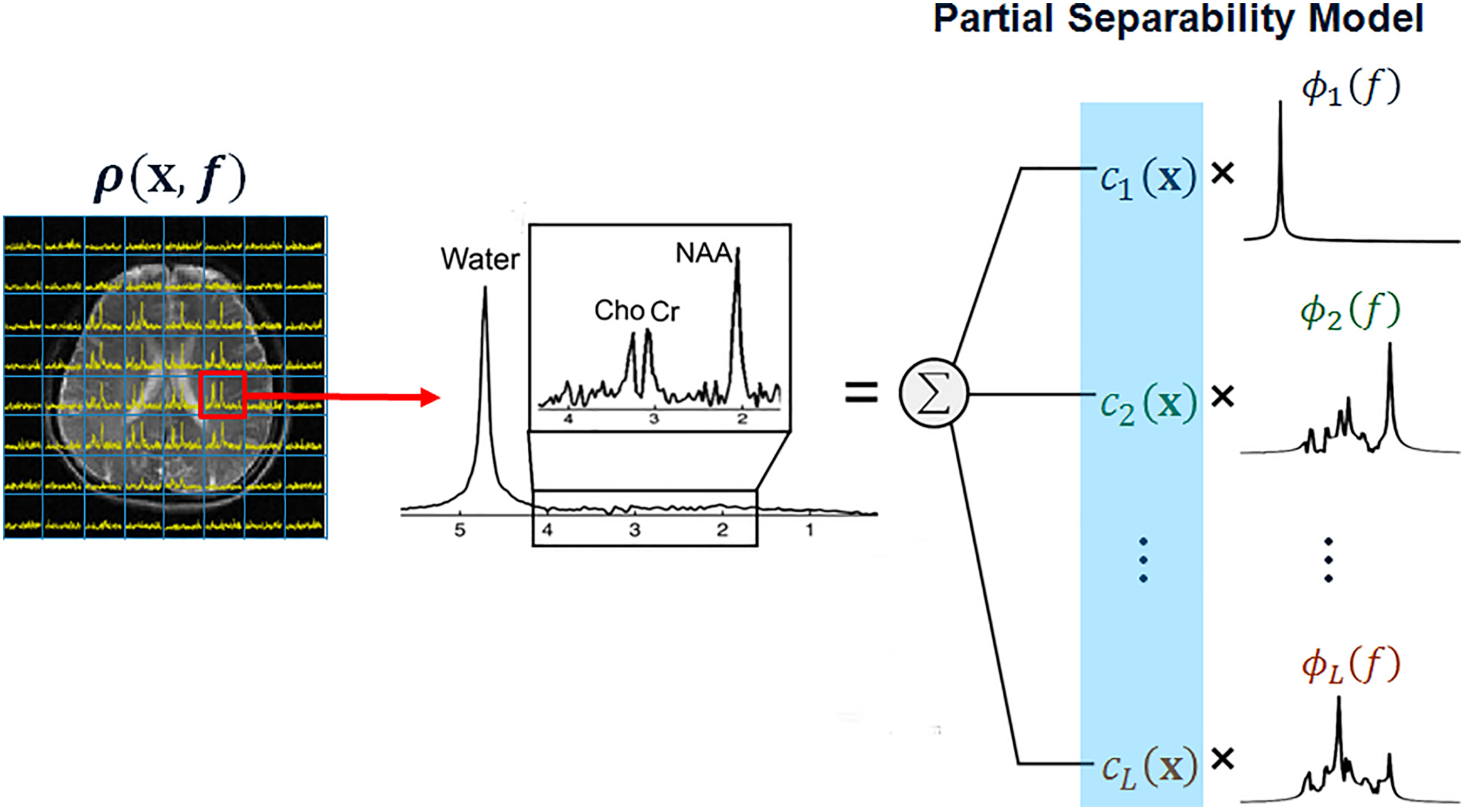
An illustration of the SPICE approach. Each voxel spectra in the high‐dimensional spatiospectral function of interest (*ρ*(*x*,*f*), the image on the left) is modeled as a linear combination of a small number of spectral basis functions $({\left\{\varphi_{l}\left(f\right)\right\}}_{l=1}^{L}$, rightmost column). This implies that the high‐dimensional signals reside in a low‐dimensional subspace (spanned by {*φ*_*l*_(*f*)}). With the subspace predetermined (from training data), the imaging problem is transformed into the estimation of a set of spatial coefficients ( ${\left\{c_{l}\left(x\right)\right\}}_{l=1}^{L}$) with much lower dimensions than the original spatiospectral function. Different subspaces can be constructed for different signal components, i.e., water, lipids and metabolites

A recent approach to reach very high acceleration factors and SNR in MRSI is the combination of the idea of “spatiotemporal imaging with partially separable functions”^287^ that separates temporal and spatial basis vectors with spectral prior knowledge and low‐rank approximation. The resulting MRSI acceleration method SPICE (spectroscopic imaging by exploiting spatiospectral correlation)^288^ represents the high‐dimensional spectroscopic imaging data as a union or superposition of subspaces (Figure 15). In practice, four subspaces are used, including metabolites, lipids, water and macromolecules.^47^ The premise of SPICE is that the existence of spatial and temporal correlations will result in a low‐dimensional representation given by the union of subspaces. Each subspace is represented using a low‐rank tensor, whose basis is estimated from training data given by a fully sampled high‐spectral‐resolution and low‐spatial‐resolution acquisition to capture spectral correlations (Figure S4). Once the basis sets have been determined, undersampled data acquired with high spatial and spectral resolution can be reconstructed by enforcing the pre‐computed union of subspaces model, which will remove aliasing artifacts and separate the spectroscopic imaging data into metabolites, lipids, water and macromolecules. The main advantage of SPICE is to learn an efficient model to represent spectroscopic images using training data, which goes beyond the handcrafted models used in CS and can enable access to higher spatial resolution and direct separation of nuisance signals. Recent implementations have eliminated the need for subject‐specific navigator data to facilitate practical application.^45^ Initial applications were shown for ^1^H‐MRSI of the human brain.^289^, ^290^ Simultaneous readouts of SPICE‐accelerated ^1^H‐MRSI with QSM^291^ and functional MRI^292^ of the human brain have been demonstrated. Other applications include mapping of brain macromolecules^47^ and dynamic ^31^P‐MRSI in skeletal muscle^293^ and hyperpolarized ^13^C‐MRSI.^294^ The development of using prior knowledge in image reconstruction from SLIM to SPICE is described in a recent book chapter.^295^

### 6.3 Super‐resolution reconstruction

Another acceleration approach is the use of interpolation^296^ to yield higher‐resolution metabolite maps from lower‐resolution MRSI data. The most simple approach is *k*‐space zero filling.^10^ Alternatively, the SENSE reconstruction framework uses additional spatial information on coil sensitivity to yield super‐resolution MRSI data.^55^, ^192^, ^193^, ^197^, ^198^ A recently presented sophisticated interpolation method is the patch‐based super‐resolution approach that uses image prior knowledge to enforce smooth transitions inside a tissue compartment and sharp tissue boundaries in interpolated metabolite images. It assumes the existence of image redundancy in the form of metabolically identical patches of voxels and thus compartments. It was applied to enhance ^1^H‐MRSI of multiple sclerosis^60^ and glioma patients.^49^ For hyperpolarized ^13^C‐MRSI super‐resolution reconstruction in the spatiotemporal dimension was proposed in combination with an alternative spatial encoding without the necessity for time‐varying SSE gradients.^297^, ^298^, ^299^ Finally, deep learning in combination with semi‐synthetic training data is utilized to yield super‐resolution MRSI data.^300^

### 6.4 Spectral‐spatial excitation

Finally, another common strategy to accelerate metabolic imaging is to use prior knowledge about the resonance frequency of spectrally well separated signals for frequency‐selective excitation. This is applied especially to hyperpolarized ^13^C‐MRI by combining spectral‐spatial pulses with fast MRI readouts^286^, ^301^, ^302^, ^303^, ^304^, ^305^, ^306^, ^307^, ^308^, ^309^ but has been shown also for ^31^P‐MRI.^310^, ^311^ Using a narrow‐band spectral‐spatial pulse to excite only a single metabolite resonance line makes the need for chemical shift encoding obsolete and enables conventional fast MRI readouts such as SSFP, echo‐planar or spiral MRI that can be further accelerated via coherent/incoherent undersampling. The application of spectral‐spatial pulses to different offset frequencies in successive MRI readouts enables fast hyperpolarized ^13^C‐MRI of multiple resonance lines and even dynamic readouts for all metabolites if applied in an interleaved manner.

## 7 SUMMARY AND OUTLOOK ON EMERGING TECHNOLOGIES

In the long history of MRSI, time‐efficient data acquisition was quickly identified as one of the main obstacles to high‐spatial‐resolution whole‐organ metabolic mapping. Although a number of alternative approaches to SSE have been proposed, SSE will certainly remain the workhorse of fast MRSI encoding, especially for ^1^H‐MRSI at 3 T and hyperpolarized ^13^C‐MRSI. However, while whole‐brain MRSI today predominantly relies on different SSE strategies, it is increasingly challenged by other means of acceleration especially at UHF, where the increased SBW and spatial resolution demands have led to performance limitations for SSE with respect to speed and SNR efficiency.^63^, ^86^ These have complicated the use of purely SSE‐based MRSI on common whole‐body gradient systems, especially since the targeted spatial resolutions have gradually increased over time.^5^, ^41^ To make efficient use of the excess SNR available at UHF (e.g., by either shortening scans or increasing resolution) and to benefit from high‐resolution strategies to minimize intra‐voxel *B*_0_ inhomogeneities,^312^, ^313^, ^314^ it will become critical to not only adapt SSE strategies, but also combine them efficiently with other means of acceleration and reconstruction algorithms. This will allow the benefits of high fields to be fully exploited.^64^, ^142^, ^148^, ^190^, ^234^ As the performance of *k*‐space undersampling increases (i.e., *g*‐factors improve with higher *B*_0_) and short‐*T*_R_ FID‐MRSI remains efficient (i.e., negligible SAR and CSDE at high *B*_0_) while the efficiency of SSE (i.e., increasing SBW and spatial resolution demand for higher gradient slew rates) decrease with increasing *B*_0_, we can expect to find an optimal balance between such combinations to slowly shift away from SSE alone to additional undersampling in the future. Depending on the application, such an efficient balance will include a reasonable compromise between short *T*_R_ (increased relaxation weighting and possibly reduced spectral resolution), undersampling (increased lipid artifacts and motion sensitivity) and SSE (increased gradient imperfections and scanner drift).^184^

The addition of spatial‐spectral prior knowledge in the reconstruction algorithm appears to be another promising way to circumvent these limits.^290^, ^295^ New reconstruction algorithms with more complex models have recently become feasible due to the constant increase in performance of computational hardware and software. However, critically questioning what kind of prior knowledge and how much regularization is truly justified to avoid bias remains an important concern. In this respect, it is also expected that deep‐learning‐based reconstruction, which has shown promising results for MRI/MRSI,^45^, ^56^, ^315^, ^316^, ^317^, ^318^ will play an increasingly important role in the near future with the potential to substantially speed up the time‐demanding reconstruction process of large multi‐channel whole‐brain MRSI datasets.

Another important step in the dissemination of fast MRSI techniques will therefore also be the progress in rapid automated spectral analysis tools tailored for whole‐brain MRSI.^319^ A number of well‐established and freely available MRSI processing pipelines already exist for application studies (e.g., MIDAS package for whole‐brain EPSI+GRAPPA; a comprehensive list of open‐source MRSI processing software is provided in the appendix). However, by no means all MRSI acceleration and reconstruction approaches described herein are available as user friendly freeware tools and hence are not yet ready for larger application studies. We anticipate that it is just a matter of time for a larger number of open‐source MRSI packages and full scanner integration of advanced MRSI methods to become available. To accelerate this process the developer community is encouraged to make MRSI sequences, reconstruction pipelines, and processing and visualization tools freely available to the scientific community. A more widespread dissemination of state‐of‐the art MRSI methodology is also needed for cross‐validation of different approaches.

Although several attempts to compare different MRSI acceleration strategies—mostly via simulations rather than experimental validation—are documented,^74^, ^86^, ^216^, ^320^, ^321^, ^322^, ^323^ a comprehensive comparison of all major SSE trajectories and undersampling techniques is not straightforward and has so far not been performed.

Considering the different and frequently hardware‐specific artifact behavior of different encoding approaches, simulations alone can only give rough guidelines. In addition, the choice of specific reconstruction pipelines and parameters have a major influence on the resulting data quality and novel forms of reconstruction (e.g., based on neuronal networks or comprehensive low‐rank models) may shift advantages/disadvantages in favor of currently more artifact‐prone, but otherwise more efficient, acquisition schemes. Hence only a qualitative comparison of the major categories of MRSI acceleration methods is provided in Table 1. It includes important considerations such as acceleration factor, SNR efficiency, typical artifacts and limitations, and suggests typical applications for each method. It is important to mention that the maximum possible acceleration factors that a particular acceleration method can achieve (Table 1) cannot be fully exploited in the case of SNR limitations. Another important factor to consider is the SRF that describes the source of the signal displayed in a given voxel. In particular, non‐Cartesian *k*‐space sampling and incoherent *k*‐space undersampling easily allow tailoring of the *k*‐space sampling density pattern (compare Table 1) and thus shaping of the SRF already during the acquisition. This allows trading the effective spatial resolution against higher SNR and better nuisance signal suppression (i.e., lipids in ^1^H‐MRSI). SRF optimization is still possible during the reconstruction, but comes at the cost of SNR efficiency.

In line with the approach taken herein, a recent consensus effort on ^1^H‐MRSI of the human brain^324^ also includes only qualitative guidelines on the application‐dependent choice of MRSI sequence parameters and describes minimal standards for a wide range of influence factors including calibration, data acquisition, quantification and visualization. This is in contrast to a recent consensus on the standardized use of semi‐LASER for clinical single‐voxel MRS at 3 T, where the benefits over PRESS are unquestionable.^325^ For MRSI, currently well‐established Cartesian SSE methods with standard *k*‐space undersampling and multi‐slice/3D options are strongly encouraged for clinical application.^324^ However, this is certainly only the most practical short‐term solution, since multi‐vendor scanner integration is already available for these methods, which is critical for clinical acceptance. The application of different encoding strategies on standardized MRSI phantoms or the same group of people in multi‐center trials is needed to provide valuable experimental evidence that will help to come up with a true consensus on which MRSI acceleration methods should be implemented by manufacturers for future clinical use.

The development of methods with both time‐efficient acquisition and reconstruction is, therefore, still ongoing and equally important for enabling clinical implementation of high‐resolution whole‐brain MRSI (or large‐organ coverage), and possibly time‐resolved MRSI such as for hyperpolarized and functional MRSI.

## FUNDING INFORMATION

The preparation of this manuscript was supported by the Austrian Science Fund (FWF)(projects KLI 718 and P 30701), the ERC starting grant 679927 / SYNAPLAST MR, Horizon 2020 ‐ H2020‐EU.3.1.6. / grant 634541 / CDS_QUAMRI and CPRIT established investigator award RR180056.

## Supporting information

Figure S1: K‐space trajectories of EPSI, spiral and concentric rings spectroscopic imaging: the arrows illustrate the readout directions for both symmetric EPSI and flyback EPSI; for symmetric EPSI, we use different‐colored arrows to differentiate the odd/even echoes for reconstruction. Reproduced from Jiang et al.^95^

Figure S2: a. Measured radial EPSI k‐space trajectories for eight projection angles (different colors). b. Magnified view of k‐space origin for selected echoes (1, 16, and 32), showing unit Δk circle reference. Sampling errors are small with respect to Δk. c. Distance from k‐space trajectory to origin for all echoes. Note that measured trajectories from late echoes contain additional noise due to T2* relaxation. Reproduced from Ramirez et al.^97^

Figure S3: EPSI readout with (a) 2D‐SENSE undersampling scheme for 2 × 2 acceleration (black = acquired, grey = non‐acquired). (b) NAA concentration maps using LCModel spectral fitting for the fully‐sampled data (8 min scan time) and 2 × 2 accelerated data (2 min scan time). (c) Spectrum (black) and LCModel fit (red) corresponding to a white‐matter voxel. Reproduced from Otazo et al.^192^

Figure S4: SPICE (SPectroscopic Imaging by exploiting spatiospectral CorrElation) sequence for 3D encoding of ^1^H‐MRSI: (a) the dual‐density, dual‐speed EPSI sequence with the slow EPSI component (left) to acquire *D*_1_and the rapid EPSI component to acquire *D*_2_ (right). Acquiring these complimentary data sets allows for determining the subspace with its spectral information and the spatial information with high spectral resolution and high SNR. τ denotes the timing for the echo shifts that can be used for additional spectral encodings; (b) the corresponding (k, t)‐space trajectories generated by the sequence in (a).^292^

## APPENDIX A

### Open‐source MRS/MRSI processing tools (in alphabetical order).

BrICS (https://brainimaging.emory.edu/brics/)—mainly display of metabolic maps and spectra.
CSItools (https://hci.iwr.uni-heidelberg.de/hci/softwares/csitools)—MRSI processing and display.
FID‐A (https://github.com/CIC-methods/FID-A)—simulation and processing of MRS(I) data.
Gannet (http://www.gabamrs.com/)—edited MRS processing.
INSPECTOR (http://innovation.columbia.edu/technologies/cu17130_inspector)—simulation and processing of MRS(I) data.
jMRUI (http://www.jmrui.eu/)—simulation, processing and display of MRS(I) data.
jSIPRO (https://www.sites.google.com/site/jsiprotool/)—processing and display of MRSI data.
MIDAS (http://mrir.med.miami.edu:8000/midas/)—acquisition, processing and display of whole‐brain MRSI data.
MRSItoolbox (https://cds-quamri.eu/index.php)—processing and display of MRSI data.
OXSA (https://github.com/oxsatoolbox/oxsa)—processing of MRS(I) data.
ProFit (https://mrtm.ethz.ch/research/mr-spectroscopy/physiological-projects/profit.html)—quantification of MRS(I) data.
SIVIC (http://sourceforge.net/projects/sivic/)—processing and display of MRS(I) data.
TARQUIN (http://tarquin.sourceforge.net/)—processing and display of MRSI data.
