# Supplementary figures and images for: Accelerated MR spectroscopic imaging—a review of current and emerging techniques
Source: NMR Biomed. 2020 May 12;34(5):e4314. doi: 10.1002/nbm.4314 (PMC8244067; doi:10.1002/nbm.4314)

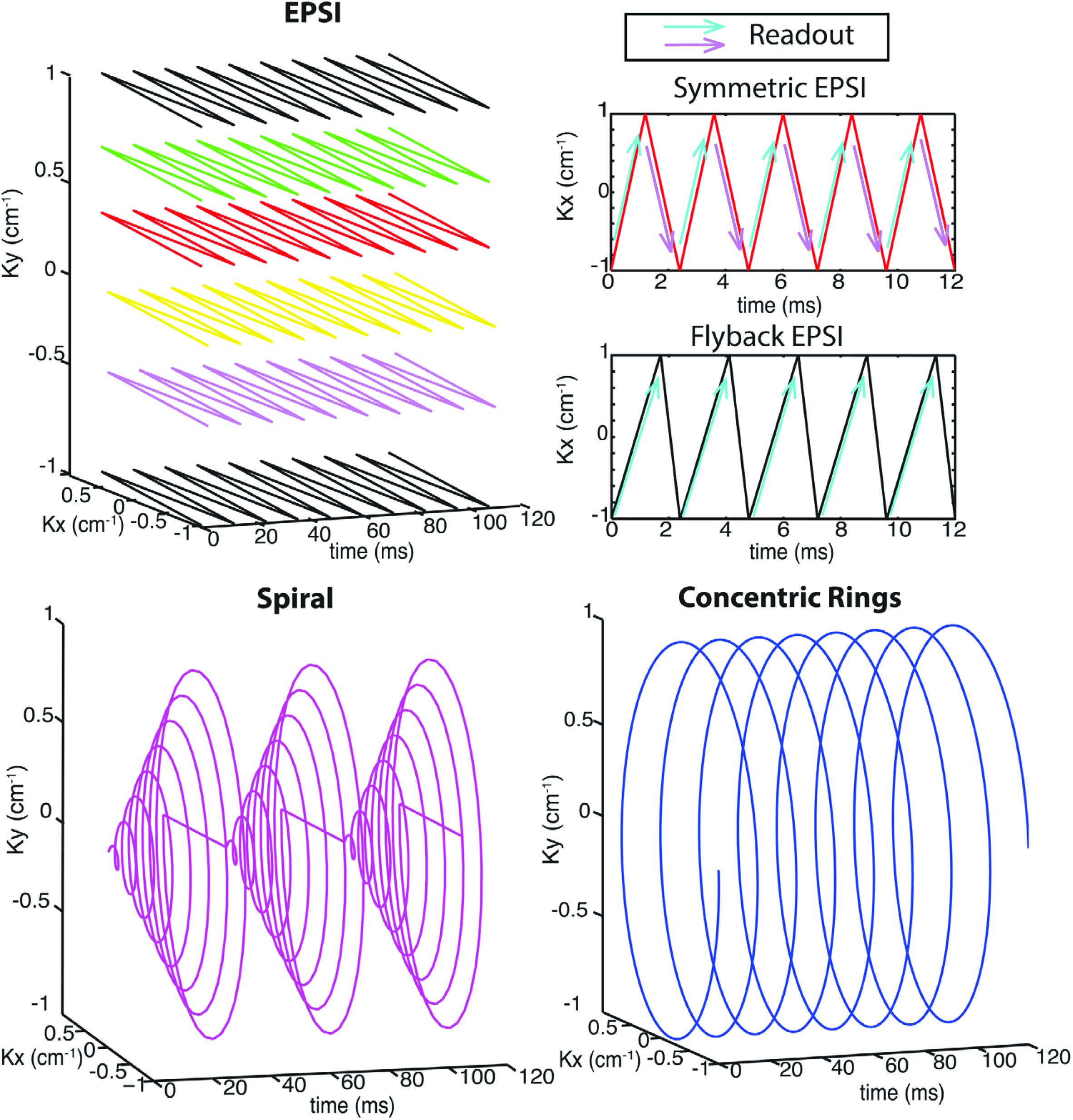

Supplement: Supplementary file 1 — Figure S1: K‐space trajectories of EPSI, spiral and concentric rings spectroscopic imaging: the arrows illustrate the readout directions for both symmetric EPSI and flyback EPSI; for symmetric EPSI, we use different‐colored arrows to differentiate the odd/even echoes for reconstruction. Reproduced from Jiang et al.95 [file NBM-34-e4314-s003.tif]

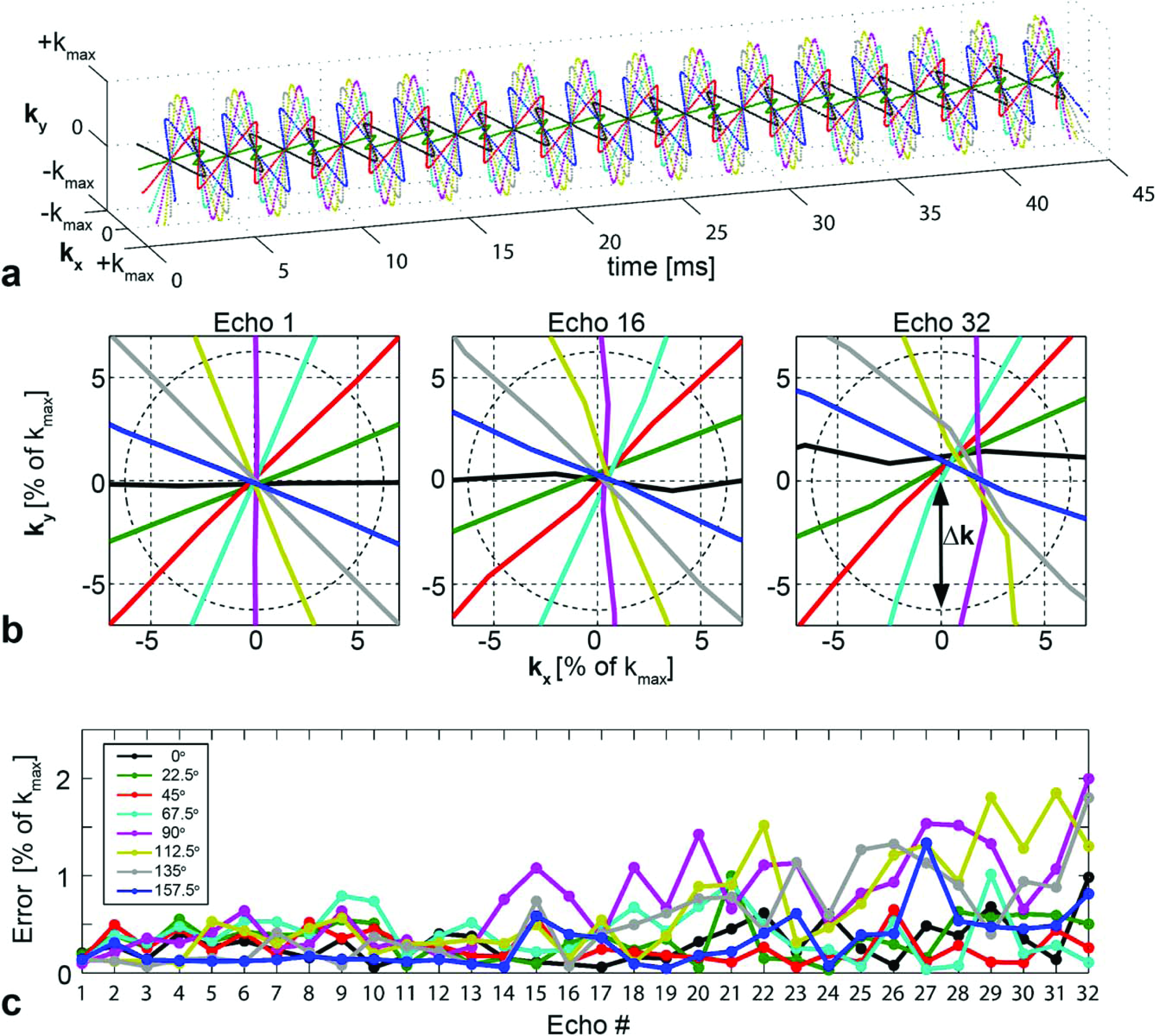

Supplement: Supplementary file 2 — Figure S2: a. Measured radial EPSI k‐space trajectories for eight projection angles (different colors). b. Magnified view of k‐space origin for selected echoes (1, 16, and 32), showing unit Δk circle reference. Sampling errors are small with respect to Δk. c. Distance from k‐space trajectory to origin for all echoes. Note that measured trajectories from late echoes contain additional noise due to T2* relaxation. Reproduced from Ramirez et al.97 [file NBM-34-e4314-s002.tif]

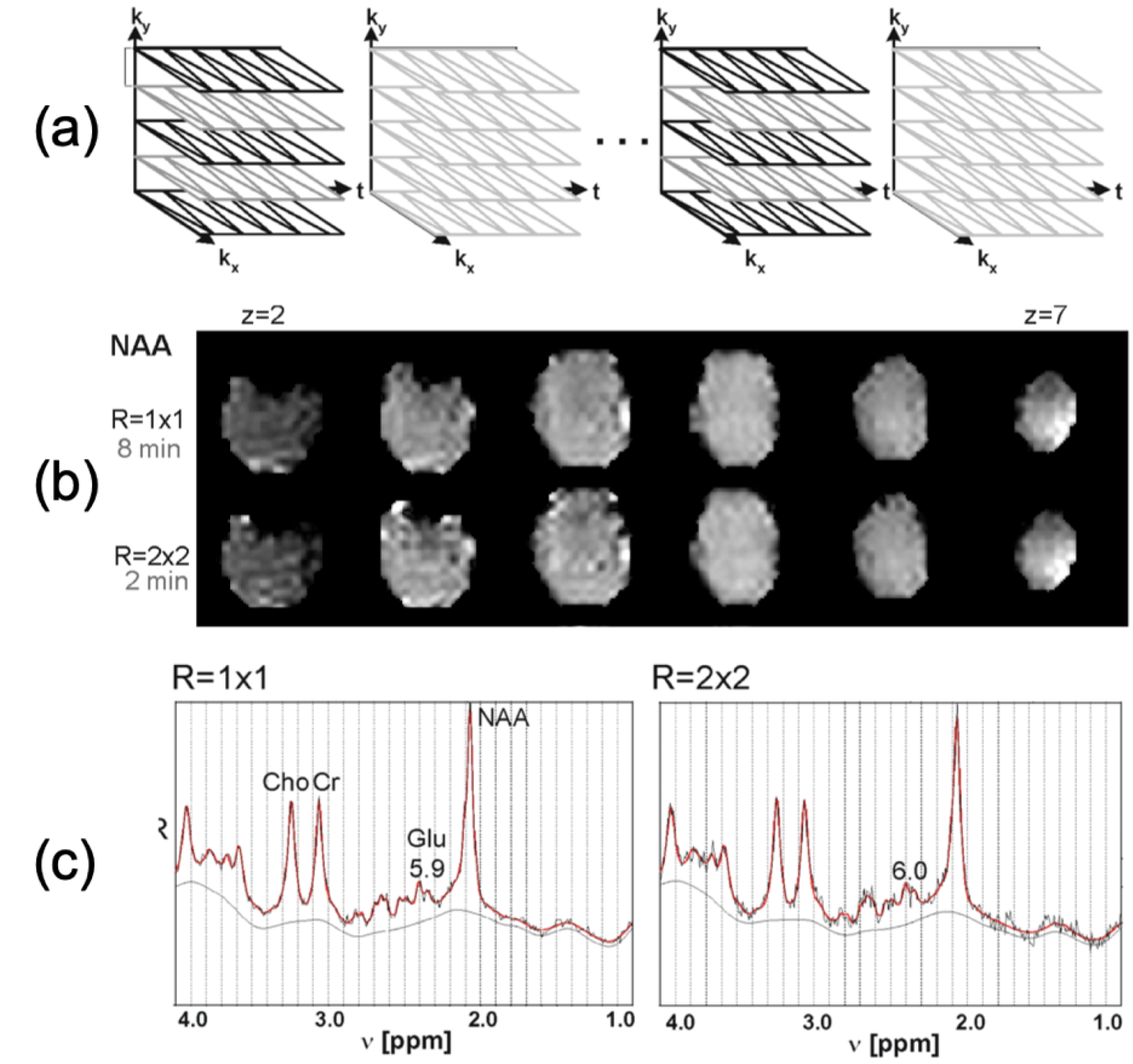

Supplement: Supplementary file 3 — Figure S3: EPSI readout with (a) 2D‐SENSE undersampling scheme for 2 × 2 acceleration (black = acquired, grey = non‐acquired). (b) NAA concentration maps using LCModel spectral fitting for the fully‐sampled data (8 min scan time) and 2 × 2 accelerated data (2 min scan time). (c) Spectrum (black) and LCModel fit (red) corresponding to a white‐matter voxel. Reproduced from Otazo et al.192 [file NBM-34-e4314-s004.tif]

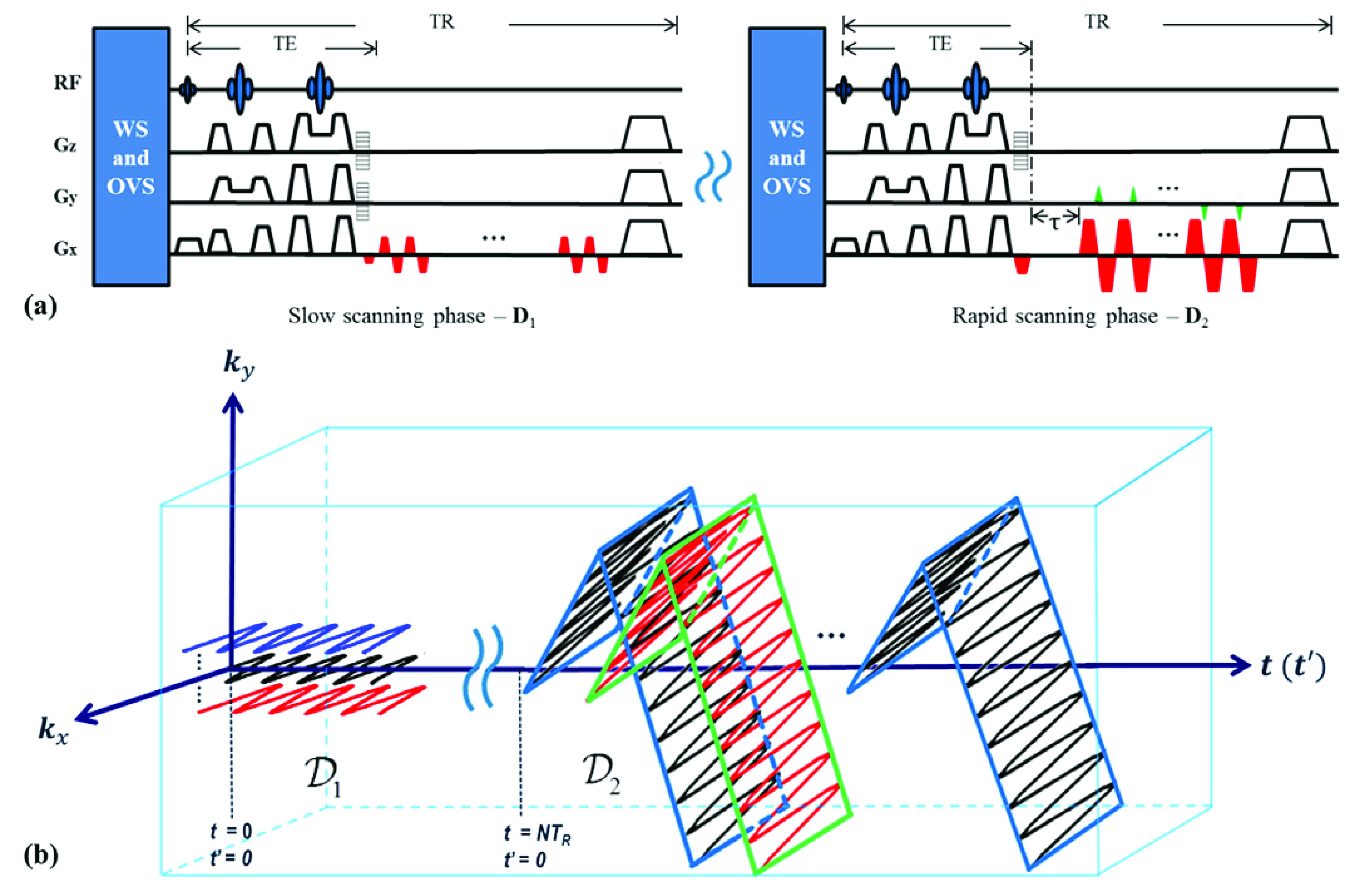

Supplement: Supplementary file 4 — Figure S4: SPICE (SPectroscopic Imaging by exploiting spatiospectral CorrElation) sequence for 3D encoding of 1H‐MRSI: (a) the dual‐density, dual‐speed EPSI sequence with the slow EPSI component (left) to acquire D 1and the rapid EPSI component to acquire D 2 (right). Acquiring these complimentary data sets allows for determining the subspace with its spectral information and the spatial information with high spectral resolution and high SNR. τ denotes the timing for the echo shifts that can be used for additional spectral encodings; (b) the corresponding (k, t)‐space trajectories generated by the sequence in (a).292 [file NBM-34-e4314-s001.tif]
